# A Novel Ultra-Stable, Monomeric Green Fluorescent Protein For Direct Volumetric Imaging of Whole Organs Using CLARITY

**DOI:** 10.1038/s41598-017-18045-y

**Published:** 2018-01-12

**Authors:** Daniel J. Scott, Natalie J. Gunn, Kelvin J. Yong, Verena C. Wimmer, Nicholas A. Veldhuis, Leesa M. Challis, Mouna Haidar, Steven Petrou, Ross A. D. Bathgate, Michael D. W. Griffin

**Affiliations:** 1 The Florey Institute of Neuroscience and Mental Health, Parkville, Victoria 3052 Australia; 2 Department of Biochemistry and Molecular Biology, University of Melbourne, Parkville, Victoria 3010 Australia; 3 IBM Research, Southbank, Victoria 3006 Australia; 4 Drug Discovery Biology, Monash Institute of Pharmaceutical Sciences, Monash University, Parkville, Victoria 3052 Australia; 5 ARC Centre of Excellence in Convergent Bio-Nano Science and Technology, Monash University, Parkville, Victoria 3052 Australia; 6 Bio21 Molecular Science and Biotechnology Institute, University of Melbourne, Parkville, Victoria 3010 Australia

## Abstract

Recent advances in thick tissue clearing are enabling high resolution, volumetric fluorescence imaging of complex cellular networks. Fluorescent proteins (FPs) such as GFP, however, can be inactivated by the denaturing chemicals used to remove lipids in some tissue clearing methods. Here, we solved the crystal structure of a recently engineered ultra-stable GFP (usGFP) and propose that the two stabilising mutations, Q69L and N164Y, act to improve hydrophobic packing in the core of the protein and facilitate hydrogen bonding networks at the surface, respectively. usGFP was found to dimerise strongly, which is not desirable for some applications. A point mutation at the dimer interface, F223D, generated monomeric usGFP (muGFP). Neurons in whole mouse brains were virally transduced with either EGFP or muGFP and subjected to Clear Lipid-exchanged Acrylamide-hybridized Rigid Imaging/Immunostaining/*In situ* hybridization-compatible Tissue-hYdrogel (CLARITY) clearing. muGFP fluorescence was retained after CLARITY whereas EGFP fluorescence was highly attenuated, thus demonstrating muGFP is a novel FP suitable for applications where high fluorescence stability and minimal self-association are required.

## Introduction

Fluorescent proteins (FPs) are versatile tools in cell biology that enable direct intracellular localisation of target proteins, indirect measurement of gene expression, measurement of protein-protein interactions or oligomeric states (in cells or in purified preparations), and can be used as biosensors for cellular signalling^1^. Green fluorescent protein (GFP) in particular, has become ubiquitous in laboratories around the world. GFP is a relatively small, inert, non-toxic globular protein that readily diffuses throughout cells and can be genetically encoded allowing non-invasive fluorescence visualization of target cells or proteins in live and fixed samples. Importantly, GFP requires only molecular oxygen to form the chromophore without the need for other cofactors^2^. More than 20 years of genetic and biochemical characterisation have led to the development of improved GFP variants with diverse spectral properties, improved quantum efficiency and greater stability to expand the versatility of FPs in cellular biology^3^. One caveat for the use of FPs is that most native FPs have a tendency to oligomerise^4–7^. Oligomerisation and aggregation of FPs may lead to undesirable experimental artefacts *in vivo*, including incorrect cellular localization^8^, reorganisation of organelles^9^ and false-positives in resonance energy transfer assays^7^. Therefore, it is important to ensure that FPs are inert fusion partners, especially when localised to complex, crowded environments such as the cell membrane^10^. While the use of FPs in biological research continues to expand, the evolving technological landscape of biological imaging is pushing the limits of currently available FP variants.

Recent advances for optically clearing large tissue sections and whole organs are revolutionising fluorescent microscopy, particularly in the field of neuroscience^11^. Techniques such as: Sca*l*e^12^; three-dimensional imaging of solvent cleared organs (3DISCO)^13^; Clear Lipid-exchanged Acrylamide-hybridized Rigid Imaging/Immunostaining/*In situ* hybridization-compatible Tissue-hYdrogel (CLARITY)^14^; and passive CLARITY^15,16^ can render whole rodent brains transparent and enable the reconstruction of neuronal circuits. To achieve clearing of fixed tissues each of these methods involves chemical and physical treatments that are often denaturing to FPs. These include: 4 M urea and Triton X-100 for several weeks in Sca*l*e^12^; tetrahydrofuran and dibenzyl ether in 3DISCO^13^; 8% sodium dodecyl sulfate (SDS), 50 °C and electrophoresis in CLARITY^14^ and; 8% SDS and 50 °C incubation for up to several weeks in passive CLARITY^15^. With the exception of Sca*l*e, each of these techniques results in the quenching of expressed FP fluorescence, necessitating the use of immunofluorescent labels^17^. Homogenous antibody penetration and specific labelling in large cleared samples is not straight forward^11^ and thus, FP variants that maintain maximal emission after the clearing process would be highly advantageous, especially in samples with low FP expression.

Recently, we applied Cellular High-throughput Encapsulation, Solubilisation and Screening (CHESS)-based directed evolution to superfolder GFP (sfGFP) and identified a GFP variant, termed ultra-stable GFP (usGFP), with superior stability when subjected to SDS and high temperatures^18^. The resistance of usGFP to long exposure times in SDS at temperatures over 50 °C makes this FP perfectly suited to the CLARITY technique. Here, to determine the suitability of usGFP for *in vivo* expression and imaging of cleared tissues, we first solved the crystal structure of usGFP to investigate the mechanism of structural stabilisation. The structure revealed a large intermolecular interface, which mediated significant self-association of usGFP. A point mutation at this interface was introduced to generate a monomeric ultra-stable GFP (muGFP) variant. Solution biophysical characterisation of muGFP showed that it has a greatly decreased tendency to self-associate with respect to usGFP, sfGFP, and enhanced GFP (EGFP), but retained high thermostability in the presence of SDS. To test the performance of muGFP *in vivo*, recombinant adeno-associated viruses (rAAV) encoding muGFP, or EGFP, were injected into the primary somatosensory cortex of mice to transduce expression of these FPs in neurons. muGFP exhibited slightly higher fluorescence staining and intensity to EGFP in transduced neurons before tissue clearing and after clearing of 2 mm sections with Sca*l*e. After clearing of 3 mm sections and whole brains with passive CLARITY however, muGFP retained high fluorescence whereas EGFP fluorescence was significantly attenuated. This work presents the development and analysis of a versatile new FP that is amenable to techniques employing denaturing conditions, such as those used for whole organ clearing.

## Results

### The crystal structure of usGFP

**Figure 1:**
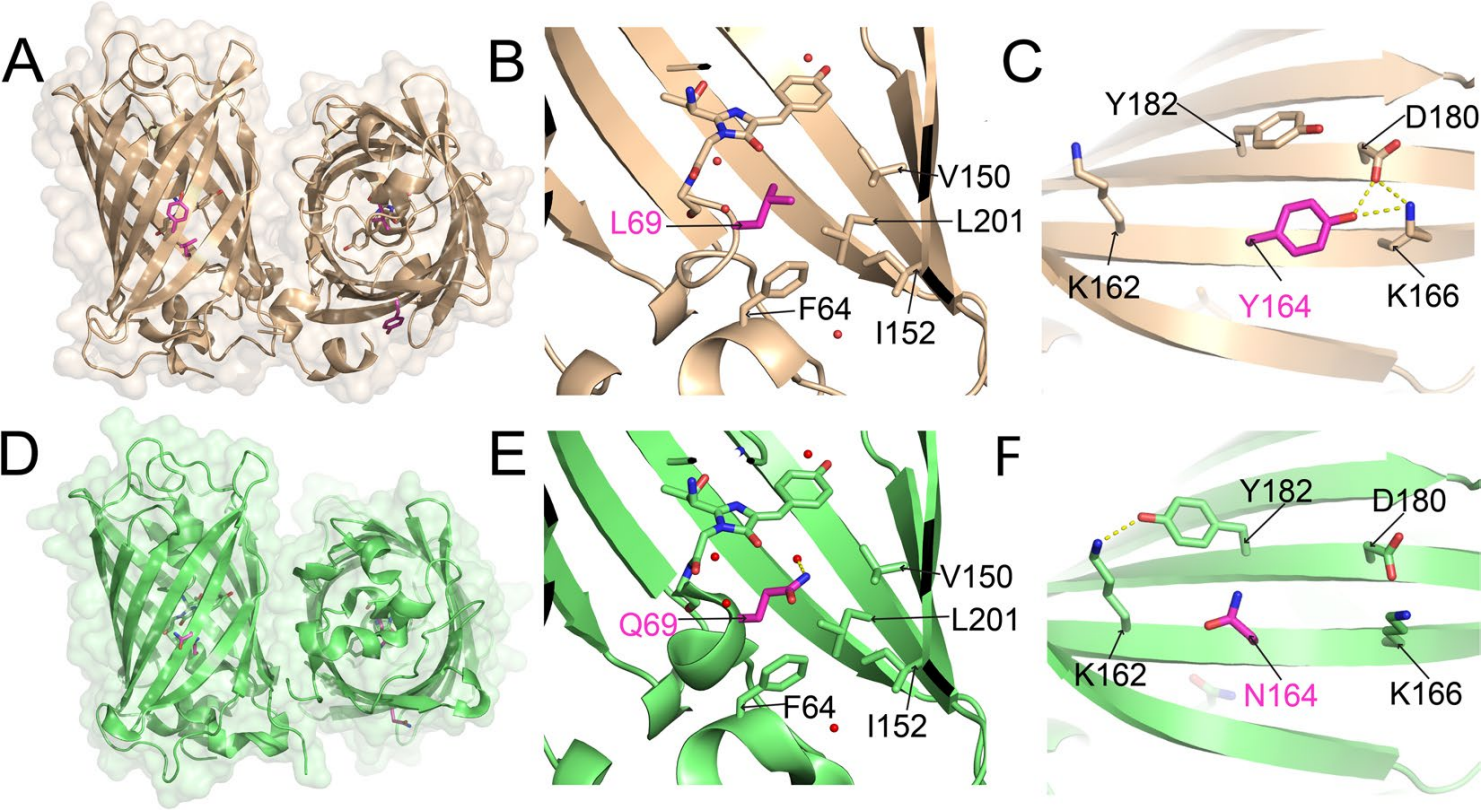
The crystal structures of sfGFP and usGFP. (**A**) Crystal structure of usGFP (orange; PDB ID: 5JZK) showing the dimeric crystallographic asymmetric unit. The chromophore is shown in stick representation and L69 and Y164 are shown in magenta. (**B**) L69 of usGFP showing the absence of the water molecule observed near Q69 of sfGFP. (**C**) Y164 of usGFP. (**D**) Crystal structure of sfGFP (green; PDB ID: 2B3P^21^). The dimer was reconstructed by crystallographic symmetry. The chromophore is shown in stick representation and Q69 and N164 are shown in magenta. (**E**) Q69 of sfGFP forms an H-bond to an ordered water molecule in the core of the structure. (**F**) N164 of sfGFP. Hydrogen bonds are shown as dashed yellow lines.

usGFP contains two point mutations, Q69L and N164Y, with respect to its parent construct, sfGFP^19^. The crystal structure of usGFP was solved at 1.9 Å resolution in order to probe the structural basis of increased stability (Table 1). Two usGFP molecules were present in the asymmetric unit (Fig. 1A) and each showed the expected 11-stranded β-barrel structure with an internal coaxial α-helix preceding the fluorophore. L69 of usGFP was situated in the core of the β-barrel, making direct contact with the chromophore. The side chain at position 69 in both usGFP (Fig. 1B) and sfGFP (Fig. 1E) was proximal to residues F84, V150, I152 and L201, which formed a hydrophobic pocket within the β-barrel. Thus, improved hydrophobic packing in this region due to the Q69L mutation may be responsible, in part, for the increased stability of usGFP. In sfGFP, a water molecule occupies a cavity in the vicinity of the Q69 side chain within the protein (Fig. 1E). This water molecule is hydrogen bonded to the side chain amide nitrogen of Q69 and encompassed by the hydrophobic residues F84, V150, I152 and L201 (Fig. 1E). In contrast, the usGFP structure shows no electron density in the equivalent position, indicating that this water molecule is absent (Fig. 1B). The exclusion of this water molecule from the interior of the usGFP structure may be due to either reduction of the size of the cavity by introduction of the leucine side chain, reduction in the hydrophilic nature of the cavity by removal of the glutamine side chain, or reduced solvent accessibility due to altered structural dynamics in this area of the structure.

**Table 1:** X-ray data collection and structure refinement statistics for usGFP and muGFP. Values for the highest resolution shell are given in parentheses.

	usGFP	muGFP
**Data collection**		
Space group	R32:h	P2_1_
Wavelength (Å)	0.9537	0.9537
Number of images	360	720
Oscillation range per image (°)	0.5	0.5
Detector	ADSC Quantum 315r	ADSC Quantum 315r
**Cell dimensions**		
a, b, c (Å)	137.18 137.18 147.65	47.35 95.71 59.65
α, β, γ (°)	90.00 90.00 120.00	90.00 104.21 90.00
Resolution (Å)	49.22–1.90 (1.94–1.90)	49.49–1.80 (1.83–1.80)
R_sym_^†^	0.089 (1.059)	0.136 (0.869)
R_meas_^§^	0.097 (1.163)	0.158 (1.017)
R_pim_^‡^	0.040 (0.478)	0.081 (0.524)
*I*/*σI*	17.8 (2.7)	11.4 (2.2)
Total observations	466265	360861
Unique reflections	41787	47783
Completeness (%)	99.4 (98.7)	99.7 (95.3)
Redundancy	11.2 (11.4)	7.6 (7.2)
Wilson B-factor (Å^2^)	27.7	10.9
Matthews Coefficient, V_M_ (Å^3^ Da^-1^)	2.38	2.34
Solvent content (%)	48.4	47.4
**Refinement**		
Resolution (Å)	49.22–1.90 (1.95–1.90)	49.49–1.80 (1.84–1.80)
Reflections used in refinement	39749 (2902)	45306 (3236)
R_free_ reflections	2035 (136)	2450 (187)
R_work_	0.142 (0.215)	0.175 (0.260)
R_free_	0.176 (0.220)	0.218 (0.304)
Protein molecules in asymmetric unit	2	2
Protein residues	470	463
Total atoms	4175	4298
Protein (including chromophore)	3764	3698
Ligand/ion	31	4
Water	380	596
Mean B-factor (Å^2^)	34.84	17.97
Protein (including chromophore)	33.67	16.05
Ligand/ion	56.08	22.11
Water	44.74	29.81
**r.m.s. deviations**		
Bond lengths (Å)	0.013	0.015
Bond angles (°)	1.572	1.727

^†^*R*_sym_ = ∑_*hkl*_∑_*i*_|*I*_*i*_ (*hkl*) − 〈*I(hkl)*〉|/∑_*hkl*_∑_*i*_*I*_*i*_ (*hkl*). ^§^*R*_meas_ = ∑_*hkl*_ [*N*/(*N* − 1)]^½^ ∑_*i*_ |*I*_*i*_ (*hkl*) − 〈*I (hkl)*〉|/∑_*hkl*_ ∑_*i*_ *I*_*i*_ (*hkl*). ^‡^*R*_pim_ = ∑_*hkl*_ [1/(*N* − 1)]^½^ ∑_*i*_ |*I*_*i*_ (*hkl*) − 〈*I (hkl)*〉|/∑_*hkl*_ ∑_*i*_ *I*_*i*_ (*hkl*).

The N164Y mutation of usGFP was located on the outer surface of the β-barrel on β-strand 8, and participated in crystal contacts via van der Waals interactions with G232 from the C-terminal α-helix of neighbouring, symmetry related molecules. The side chain hydroxyl group of the substituted residue, Y164, of usGFP formed intramolecular hydrogen bonds with the side chains of K166 and D180 (Fig. 1C). In contrast, the sidechain of N164 of sfGFP, which was exposed to a solvent channel in the crystal, did not appear to form any strong hydrogen bonds with surrounding side chains, while the side chain amine group of neighbouring residue K162 formed a hydrogen bond with the side chain hydroxyl group of Y182 (Fig. 1F). Thus, the increased reach of the tyrosine side chain with respect to the asparagine may mediate altered or more extensive hydrogen bonding networks at the surface of the protein and, thus, contribute to structural stability of usGFP. Interestingly, the orientation of the side chains of residue 164 and Y182 mirrored each other in their respective structures.

Examination of the interface between the two molecules of the asymmetric unit of usGFP showed that this created a buried surface area of 823 Å^2^ (7.7% total surface area), determined using PISA^20^. Although sfGFP was reported to be monomeric^21^ a similar interface was found in the sfGFP crystal structure. Generation of the symmetry related molecules from the single protein molecule of the sfGFP asymmetric unit provided a dimeric structure, essentially the same as that determined for usGFP (Fig. 1D, Cα RMSD 0.688 Å), with a buried surface area of 977 Å^2^ (9.3% total surface area) at the interface.

### Solution properties of usGFP, sfGFP, and EGFP

**Figure 2:**
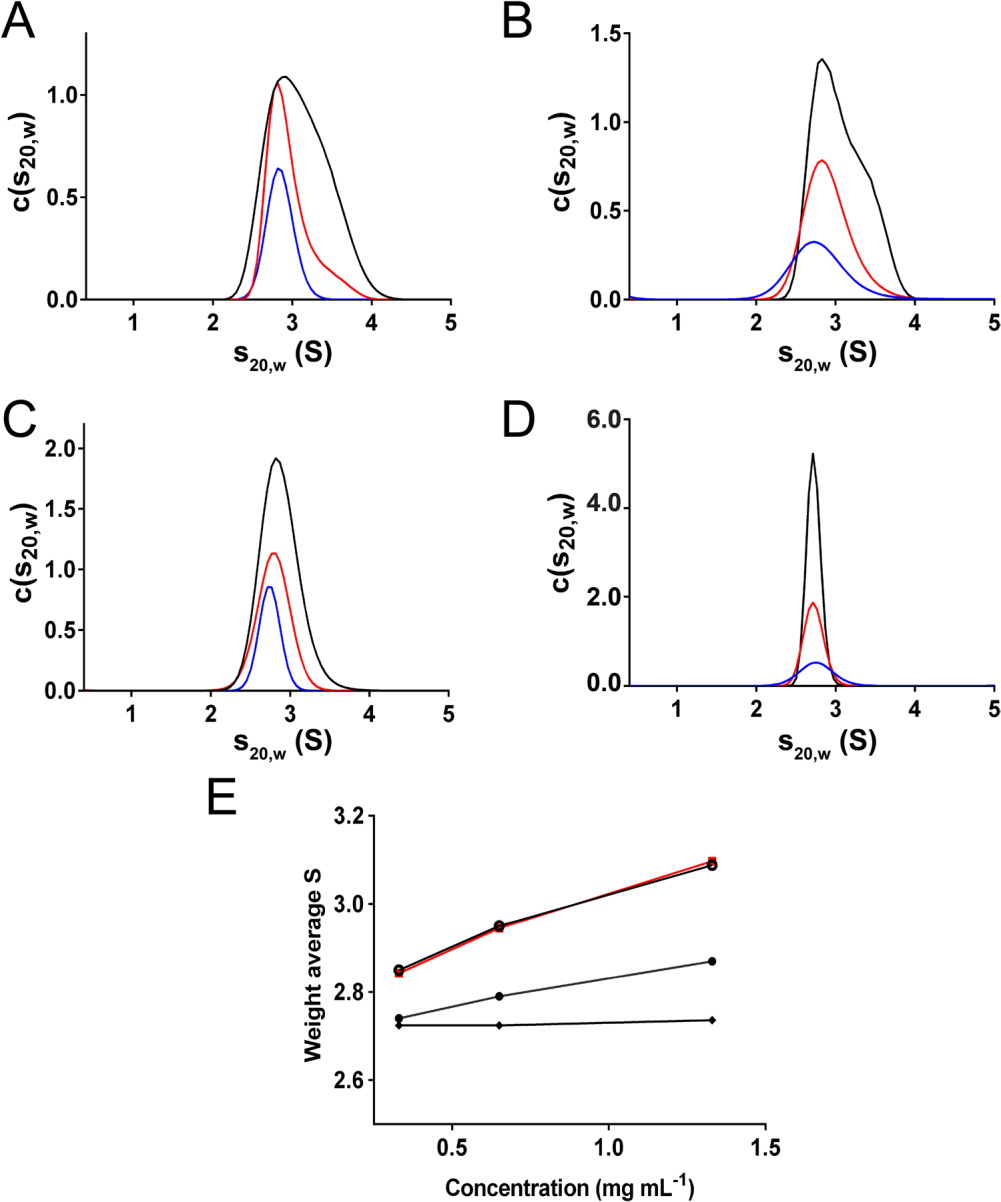
Sedimentation velocity analysis and concentration-dependent self-association of usGFP, sfGFP, EGFP and muGFP. Standardised continuous sedimentation coefficient [c(s_20_,_w_)] distributions for (**A**) usGFP, (**B**) sfGFP (**C**) EGFP and (**D**) muGFP at concentrations of 0.33 mg mL^−1^ (blue line), 0.65 mg mL^−1^ (red line) and 1.33 mg mL^−1^ (black line). (**E**) Weight average sedimentation coefficients for sfGFP (red squares), usGFP (open circles), EGFP (solid circles), and muGFP (solid diamonds), calculated from c(s_20,w_) distributions shown in panels (A–D) for usGFP, sfGFP, EGFP and muGFP. Distributions were integrated between 1 S and 5 S using SEDFIT.

As the crystal structures of usGFP and sfGFP suggested that these proteins oligomerise, a thorough biophysical characterisation of usGFP, sfGFP, and enhanced GFP (EGFP) was undertaken to determine and compare the solution properties of these GFP variants. To determine if oligomerisation was also occurring in solution, sedimentation velocity (SV) analytical ultracentrifugation (AUC) was conducted on usGFP and sfGFP. Each variant was analysed at three concentrations to examine concentration-dependent self-association (Fig. 2; Supplementary Figures 1 and 2). usGFP and sfGFP both showed broad peaks in the c(s_20,w_) distribution between approximately 2 and 4S (Fig. 2A and B) suggesting some heterogeneity in the solution oligomeric structure. Increasing protein concentration caused these distributions to skew toward higher sedimentation coefficients, suggesting concentration dependent self-association. To investigate this further, the weight average sedimentation coefficients were calculated at each protein concentration. The significant increase in weight average sedimentation coefficient as a function of concentration for both usGFP and sfGFP indicated self-association, and that the affinity of this interaction was similar for the two variants (Fig. 2E). We also performed AUC on EGFP, a commonly used variant of GFP engineered for increased brightness^22^ (Fig. 2C; Supplementary Figure 3). EGFP showed a smaller concentration dependent increase in weight average sedimentation coefficient compared to usGFP and sfGFP (Fig. 2E), indicating that this variant also displayed significant self-association.

**Figure 3:**
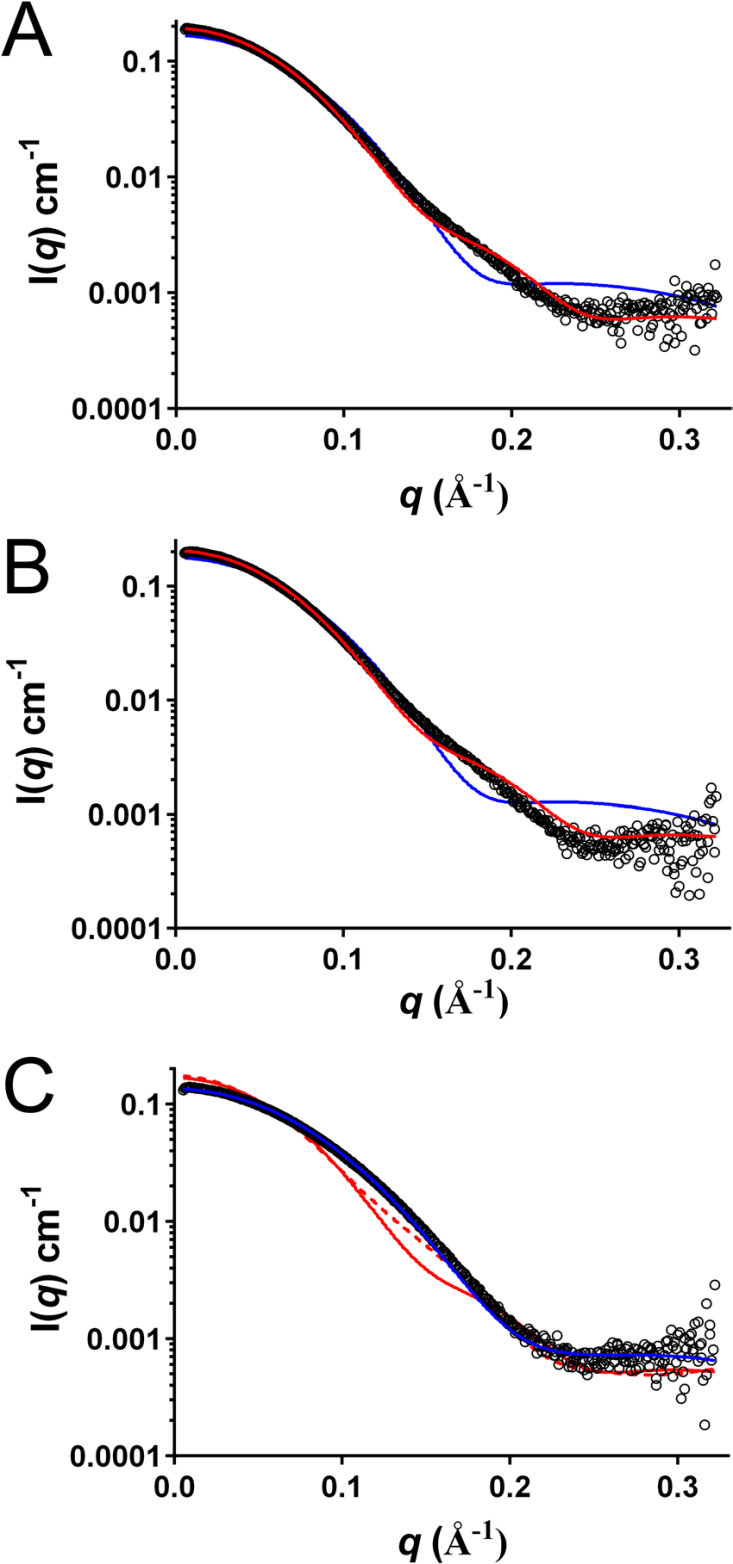
SAXS analysis of usGFP, sfGFP and muGFP. Experimental scattering data (open circles) for (**A**) usGFP (**B**) sfGFP and (**C**) muGFP are overlaid with fits to theoretical scattering profiles calculated with CRYSOL for the usGFP monomer (blue line), dimer (red line) and dimeric asymmetric unit of the muGFP crystal structure (red dotted line in (**C**)).

Small angle X-ray scattering (SAXS) was used to further characterise the oligomeric structure of usGFP and sfGFP (Fig. 3A and B). In-line size-exclusion chromatography coupled to the SAXS/WAXS beam line at the Australian Synchrotron (SEC-SAXS) was employed. usGFP and sfGFP were loaded at concentrations of approximately 50 mg mL^−1^ to ensure a high concentration (>approximately 10 mg mL^−1^) was reached after dilution on the column. In each experiment, calculated radius of gyration (R_*g*_) was stable across the elution peak, suggesting monodispersity in each sample (Supplementary Figure 4). The R_*g*_ values measured for usGFP and sfGFP were 23.77 ± 0.25 Å and 23.64 ± 0.22 Å, respectively. The experimental SAXS profiles were compared with scattering profiles calculated for the monomer and dimer of the usGFP crystal structure coordinates (Fig. 3). The experimental scattering profile for usGFP fitted significantly better to the theoretical scattering profile of the dimer (χ = 3.81) than to the monomer (χ = 10.11) (Fig. 3A). Similarly, the experimental scattering profile for sfGFP fitted well to the theoretical scattering profile of the usGFP dimer (χ = 4.11), but poorly to the scattering profile calculated for the monomer (χ = 10.88) (Fig. 3B). These data confirmed that sfGFP and usGFP existed predominantly as dimers in solution under these conditions. Small deviations of the experimental data from the profile calculated for the usGFP dimer at higher *q* values may be due to the conformation of the C-terminal α-helix, which forms intermolecular crystal contacts in the structure but does not make significant intramolecular contacts. Thus, this part of the protein may adopt a different or dynamic configuration in solution.

### The design and structure of monomeric usGFP

Oligomerisation of FPs is undesirable for some experiments, as it can introduce artefacts in systems where fusion proteins experience crowded molecular environments or are present in high concentrations. As our solution biophysical data indicated that usGFP displayed significant dimerisation, we sought to design a variant of usGFP that remains monomeric. Examination of the interface between the two monomers of the usGFP dimer revealed several hydrophobic contacts. The symmetry of the dimer placed F223 close to its counterpart in the second molecule (Supplementary Figure 5). Thus, a single mutation replacing F223 with the negatively charged amino acid aspartate (F223D) was introduced into usGFP to disrupt this interface by electrostatic repulsion of like charge, as described previously^23^ and this mutant was referred to as monomeric usGFP (muGFP).

**Figure 4:**
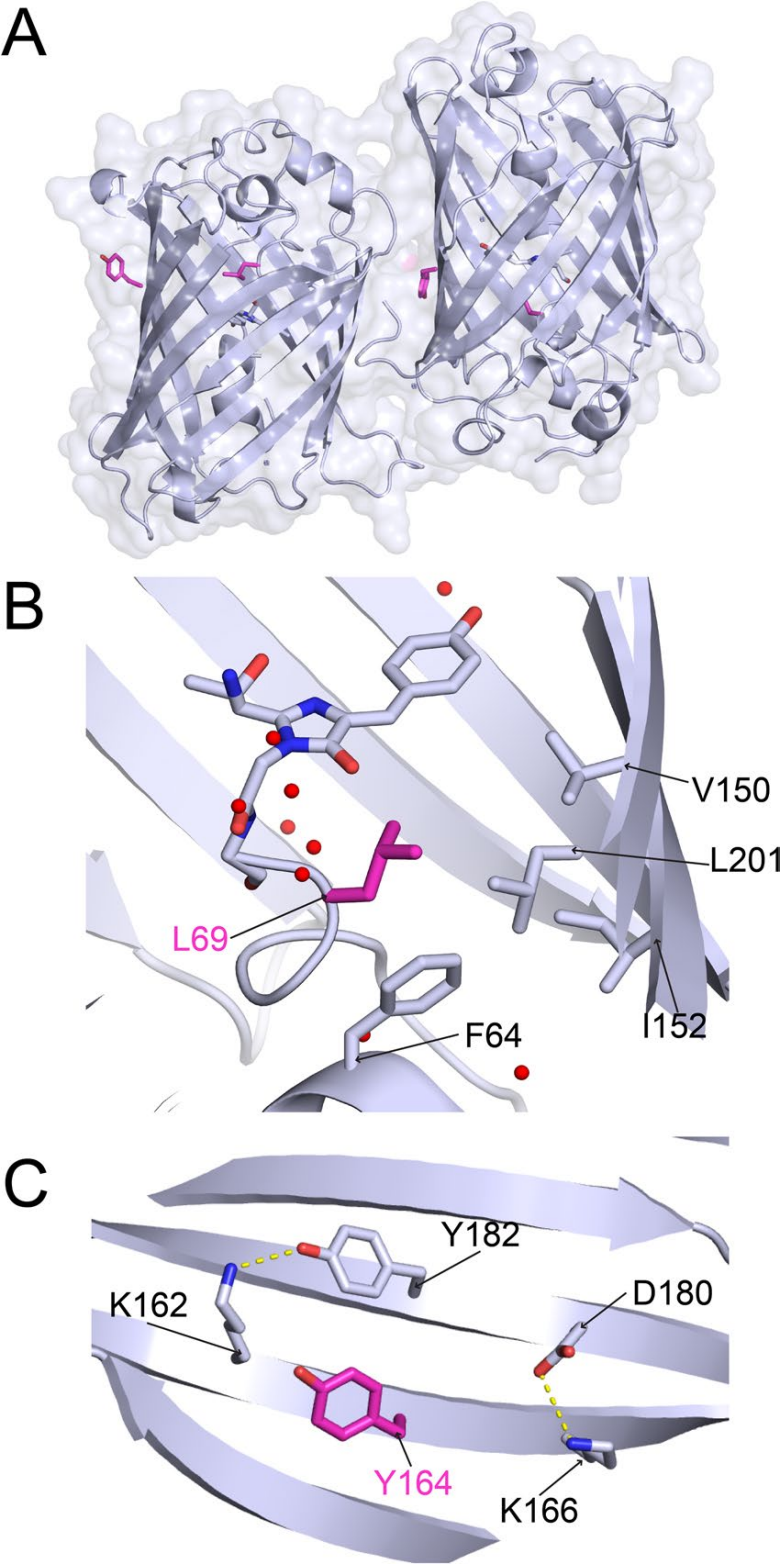
The crystal structure of muGFP. (**A**) The crystal structure of muGFP (PDB ID: 5JZL). The two molecules of the asymmetric unit were oriented with the β-barrel axes approximately parallel to one another. The chromophore is shown in stick representation and L69 and Y164 are shown in magenta. (**B**) L69 of muGFP showing the absence of the water molecule observed near Q69 of sfGFP. (**C**) Y164 of muGFP. Hydrogen bonds are shown as dashed yellow lines.

The crystal structure of muGFP was solved at 1.8 Å resolution and contained two molecules in the asymmetric unit (Fig. 4A). The crystallographic interface between these two molecules was altered in muGFP compared with usGFP, and generation of symmetry mates within the lattice failed to provide any crystal contacts that resembled the usGFP dimer interface. This suggested that the F223D mutation sufficiently disrupted the dimeric interface observed in usGFP. Examination of L69 in muGFP showed that this residue had a similar orientation to L69 of usGFP. Importantly, the water molecule observed near Q69 in sfGFP was also absent in muGFP (Fig. 4B). The crystal lattice of muGFP had changed with respect to usGFP such that Y164 in muGFP formed part of a crystal contact with the edge of the neighbouring β-barrel in all molecules. Y164 of muGFP also showed an altered rotamer with respect to Y164 of usGFP, and did not form hydrogen bonds with K166 and D180, but rather, a hydrogen bond was formed between K166 and D180 (Fig. 4C). muGFP Y182 formed a hydrogen bond with K162, which was not observed in usGFP (Fig. 4C). As observed for usGFP and sfGFP the orientations of the Y164 and Y182 side chains were similar in this structure.

To confirm disruption of the dimeric structure SV AUC experiments were conducted with muGFP as for sfGFP and usGFP (Supplementary Figure 6). The resulting c(s_20,w_) distributions showed narrow, symmetrical peaks suggesting a single homogeneous species in solution (Fig. 2D). The absence of concentration dependent increase in the weight average sedimentation coefficient (Fig. 2E) indicates the absence of self-association of muGFP in this concentration range and suggests that the F223D mutation was sufficient to prevent dimer formation. The solution structure of muGFP was further investigated using SAXS. These analyses indicated that muGFP had an R_*g*_ of 20.26 ± 0.54 Å, which is significantly lower than those of sfGFP and usGFP, suggesting a monomeric structure. Furthermore, the experimental scattering profile of muGFP fitted well to the theoretical scattering profile for the monomer (χ = 2.40) (Fig. 3C), but poorly to the theoretical scattering profile for the usGFP dimer (χ = 20.37). Further comparison of the muGFP experimental scattering profile with the calculated scattering profile of the dimer observed in the asymmetrical unit of muGFP (Fig. 3C) also showed poor agreement (χ = 18.52).

### muGFP is a bright, ultra-stable FP

**Figure 5:**
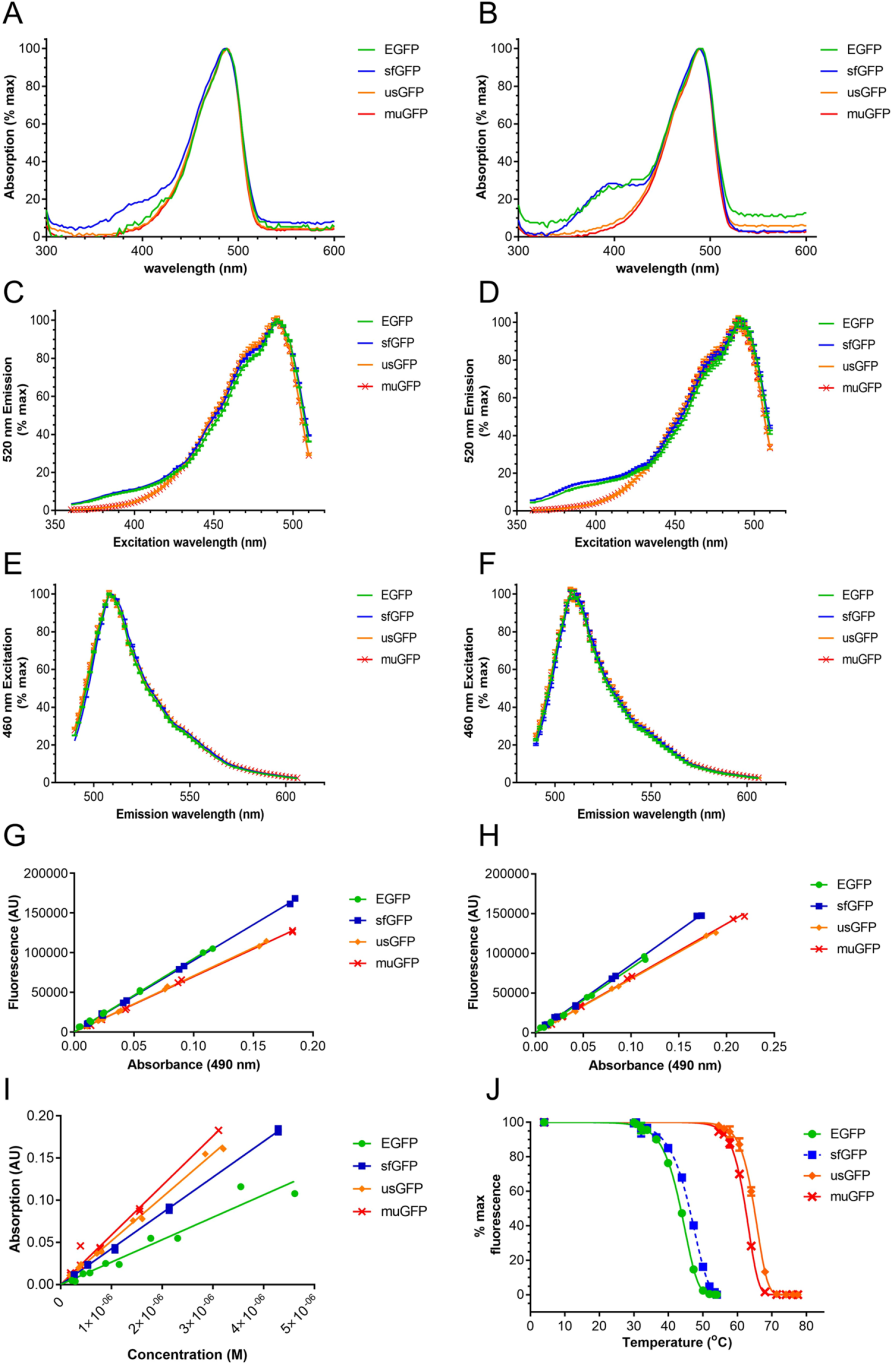
Spectral and stability characteristics of FPs. Purified EGFP (green symbols and lines), sfGFP (blue symbols and lines), usGFP (orange symbols and lines) and muGFP (red symbols and lines) were used to measure absorption, fluorescence excitation and fluorescence emission spectra in the absence (**A**,**C** and **E** respectively) and presence of 2% SDS (**B**,**D** and **F** respectively). Plotting absorbance against fluorescence for five concentrations of each FP allowed the calculation and comparison of quantum yields in the absence (**G**) and presence (**H**) of 2% SDS. Molar extinction coefficients were calculated for each variant by plotting protein concentration against absorbance at 490 nm (**I**). The fluorescence of EGFP (green solid circles and line), sfGFP (blue solid squares with dashed line), usGFP (orange diamonds with solid line) and muGFP (red crosses with solid line) was measured after incubation at the specified temperatures in the presence of 1% SDS for 30 min (**J**). The calculated apparent melting temperatures (T_m_) are listed in Table 2, and were all significantly different from each other as determined by two-way ANOVA and Tukey’s multiple comparisons test.

The fluorescence characteristics of purified EGFP, sfGFP, usGFP and muGFP were analysed under the same conditions in the absence or presence of SDS. All of the FPs exhibited the same absorption, fluorescence excitation and fluorescence emission maxima, which were unchanged in the presence of SDS (Fig. 5A–F and Table 2). Interestingly, sfGFP exhibited a second absorption shoulder at approximately 400 nm in the absence of SDS, of which the relative intensity increased in the presence of SDS, with respect to the 490 nm peak. A similar 400 nm absorption shoulder was also observed for EGFP in the presence of SDS. Correspondingly, significant fluorescence excitation was observed for EGFP and sfGFP at 400 nm in the absence and presence of SDS (Fig. 5C,D). The absorption and fluorescence spectra of usGFP and muGFP were not significantly different upon the addition of SDS. The quantum yields of usGFP and muGFP were lower than EGFP and sfGFP in the absence or presence of SDS (Fig. 5G,H and Table 2). Interestingly, whereas the addition of SDS caused a reduction in the quantum yield of EGFP and sfGFP, SDS had no effect on the quantum yields of usGFP and muGFP. Conversely the molar extinction coefficients of usGFP and muGFP were significantly higher than EGFP (Fig. 5I and Table 2), resulting in significantly higher brightness from usGFP and muGFP, especially in the presence of SDS.

**Table 2:** Characteristics of EGFP, sfGFP, usGFP and muGFP.

	EGFP	sfGFP	usGFP	muGFP
λ_ex_ (nm)	490	490	490	490
λ_em_ (nm)	508	508	508	508
ε_490nm_ (x1000)	53 ± 6.5	85 ± 1.5	103 ± 3.5	117 ± 7
QY_Buf_	0.6 (std)	0.59 ± 0.01	0.45 ± 0.01	0.45 ± 0.01
QY_SDS_	0.53 ± 0.01	0.57 ± 0.01	0.45 ± 0.01	0.45 ± 0.01
Brightness _Buf_	31.8	50.2	46.4	52.7
T_m_ (°C)	43.5 ± 0.1	46.1 ± 0.1	64.9 ± 0.1	62.3 ± 0.1
Aggregation	dimer	dimer	dimer	monomer

λ_ex_ is the excitation maximum and λ_em_ is the emission maximum. ε_490nm_ is the molar extinction coefficient at 490 nm absorption, numbers presented are the calculated extinction coefficients (M^−1^ cm^−1^) divided by 1000. Calculated quantum yields in buffer (QY_Buf_) or SDS (QY_SDS_). Brightness is ε multiplied by QY (units M^−1^ cm^−1^), divided by 1000.

The thermal stability of EGFP, sfGFP, usGFP and muGFP was tested in the presence of SDS. FPs were incubated in buffer containing 1% SDS and heated at various temperatures for 30 min before measurement of fluorescence. The apparent melting temperature, T_m_, of each protein was determined as the temperature where each sample exhibited 50% of initial fluorescence. muGFP exhibited greatly improved thermostability in SDS compared to EGFP and sfGFP (Fig. 5J and Table 2). The stability of muGFP was slightly, but significantly, reduced with respect to that of usGFP, however despite this small reduction it was clear that the dimerisation of usGFP was not a major contributing factor for the increased stability of usGFP.

Fixation is commonly used to preserve cell and tissue samples for fluorescence microscopy and is an important step preceding tissue clarification. To assess the impact of formaldehyde-based fixation on EGFP, sfGFP and muGFP fluorescence, FPs were transiently expressed in HEK-293T cells and fixed with standard fixation using 4% paraformaldehyde (PFA). The effects of the cross-linkers, 4% PFA + 5% glutaraldehyde and 4% PFA + 5% methanol, on fluorescence emission were also tested, as these are used in some fluorescence microscopy protocols. Bright-field and GFP fluorescence images show comparable transfection efficiencies for sfGFP and muGFP, and lower fluorescence loss in muGFP-expressing cells following standard fixation (4% PFA, 20 min at 4 °C) when compared to its unstabilised counterpart, sfGFP (Supplementary Figure 7A). Relative to a PBS control treatment group data shows fluorescence loss for 4% PFA in PBS (sfGFP 53.7 ± 2.3%; muGFP, 68.7 ± 3.0%; EGFP 84.4 ± 2.3%, relative to PBS control) cells exposed to 4% PFA + 5% glutaraldehyde (sfGFP 59.49 ± 2.3%; muGFP, 82.7 ± 5.8%; EGFP 69.81 ± 1.3%) and 4% PFA + 5% methanol (sfGFP 66.14 ± 2.5%; muGFP, 84.9 ± 4.6%; EGFP 89.77 ± 6.34%) (Supplementary Figure 7B). Together, muGFP outperformed sfGFP in all conditions and maintained greater fluorescence than EGFP in cells treated with 5% glutaraldehyde.

### muGFP retains bright fluorescence in cleared mouse brains

**Figure 6:**
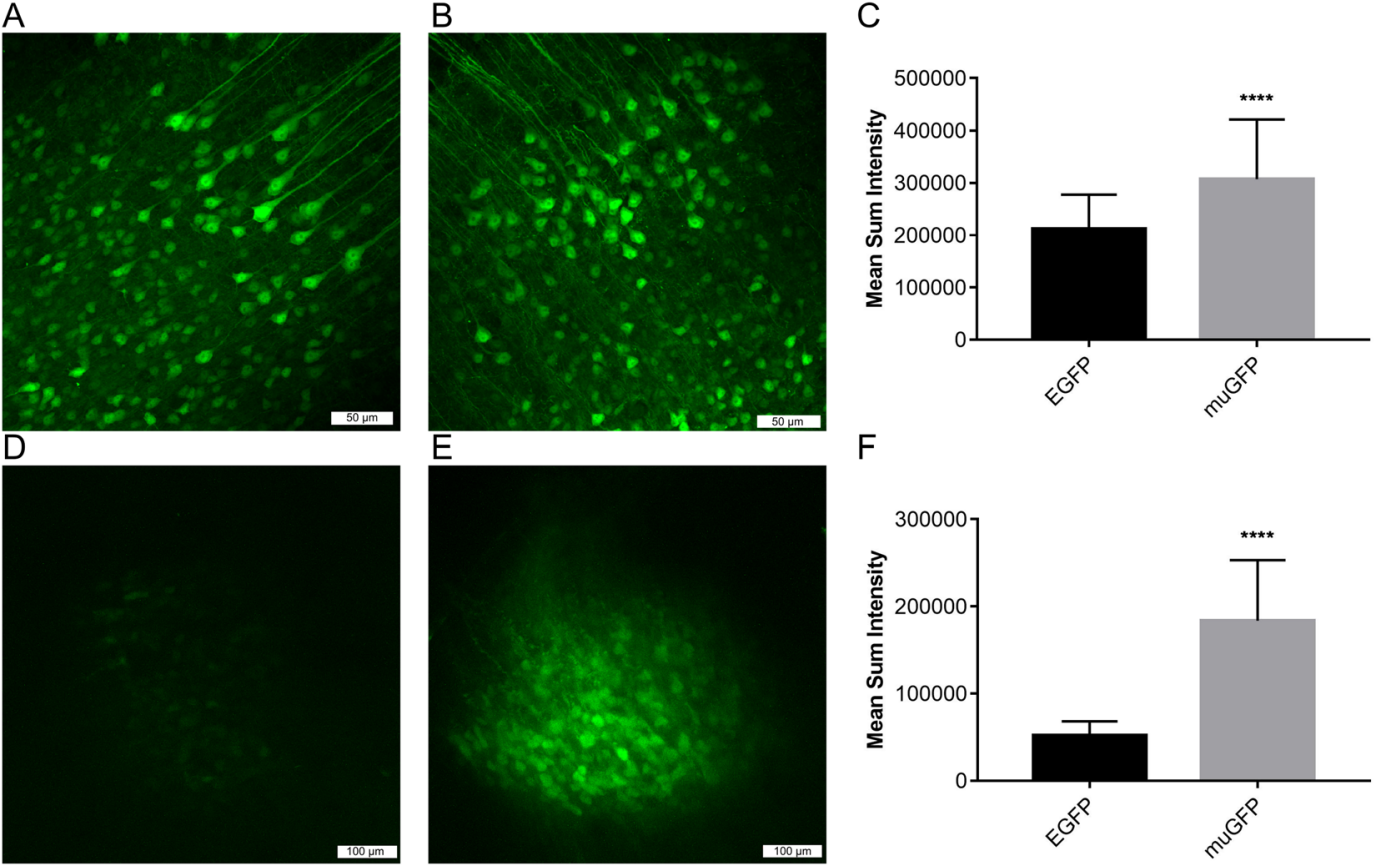
Confocal imaging and fluorescence intensity quantification of pre- and post-CLARITY cleared sections of mouse cortex expressing EGFP and muGFP. Neurons of the primary somatosensory cortex of four mice were virally transduced with EGFP in the left hemisphere (**A** and **D**) and muGFP in the right hemisphere (**B** and **E**). Maximum projections from a representative brain of confocal image stacks from a fixed coronal brain section with no clearing, with EGFP transduced neurons (**A**) exhibiting slightly less fluorescence than muGFP transduced neurons (**B**). (**C**) The mean sum intensities of transduced neurons from two brains were calculated, with muGFP exhibiting significantly higher staining intensity. Maximum projections of confocal image stacks from a CLARITY cleared 3 mm section demonstrated that EGFP fluorescence was highly attenuated (**D**) compared to muGFP (**E**), which was quantified with sections from two brains (**F**). Groups were compared using an Exact Mann-Whitney ranksum test for equality of medians and **** indicates the medians were significantly different, p < 0.05.

**Figure 7:**
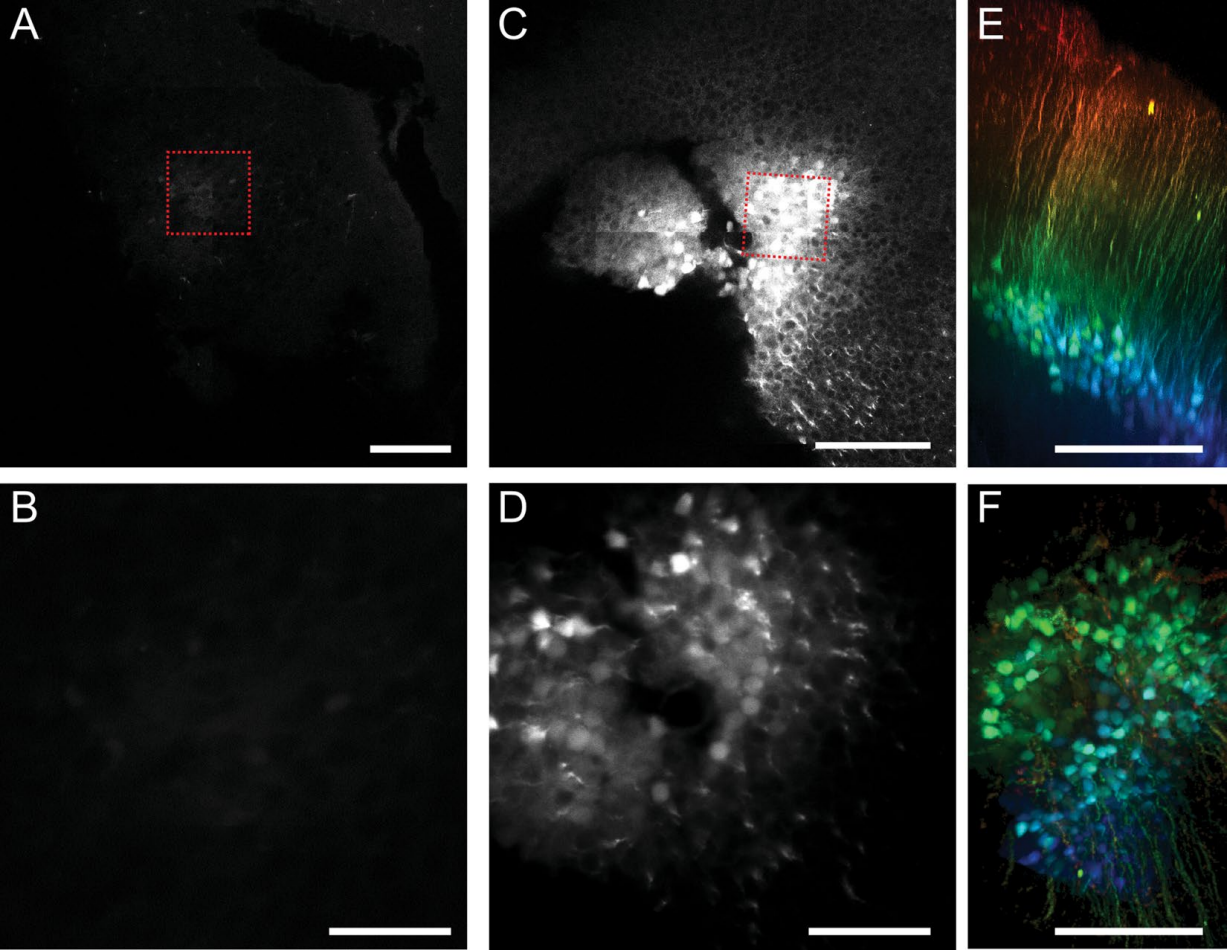
Confocal imaging of a representative CLARITY cleared whole mouse brain expressing EGFP and muGFP. The primary somatosensory cortex was virally transduced with EGFP in the left hemisphere (**A**–**D**) and muGFP in the right hemisphere (**C**–**F**) and the whole brains cleared with passive CLARITY. Cleared whole brains were imaged with confocal microscopy and maximum projections of confocal image stacks generated (images from a representative brain presented in **A**–**D**). Cortical injection sites and high magnification L5 neurons at ~750 μm depth from pia expressing EGFP (**A** and **B**) and muGFP (**C** and **D**). Red dashed squares indicate where higher magnification images were taken. 3D reconstructions of 1.4 mm confocal Z-stacks of muGFP expressing neurons in coronal (**E**) and tangential view (**F**). The stacks are coloured arbitrarily to differentiate the top from the bottom. Scalebars are (**A**–**C**) 200 μm, (**B**–**D**) 50 μm, E-F 500 μm. Replicate cleared whole brains are presented in Supplementary Figure 8.

To assess the performance of muGFP in cleared tissue samples, rAAV viral vectors were produced encoding muGFP or EGFP. To enable direct comparison, rAAV1/2-EGFP and rAAV1/2-muGFP were injected into the primary somatosensory cortex on the left and right hemispheres of each live mouse brain respectively. Two weeks after injection, mouse brains were either fixed via 4% PFA transcardial perfusion for imaging uncleared brains and for Sca*l*e-based clearing, or perfused with CLARITY hydrogel for CLARITY-based clearing. Confocal microscopy of fixed, uncleared brains revealed intense fluorescence staining of neurons transduced with both rAAV1/2-EGFP and rAAV1/2-muGFP (Fig. 6A and B), indicating muGFP was well expressed in neurons *in vivo*. The mean sum intensity of cells expressing muGFP was significantly higher than those expressing EGFP (Fig. 6C), likely due to the increased brightness of muGFP (as observed in Fig. 5). In 3 mm sections cleared with passive CLARITY (4% SDS and 55 °C for 3 weeks), EGFP fluorescence was strongly diminished, whereas muGFP transduced neurons remained brightly fluorescent (Fig. 6D–F). The loss of EGFP fluorescence was more pronounced in cleared whole brains (4% SDS and 55 °C for 5 weeks), whereas muGFP fluorescence remained bright (Fig. 7), similar to the 3 mm sections. Furthermore, the high fluorescence of muGFP in CLARITY-cleared whole brains enabled the imaging of confocal z-stacks for three-dimensional reconstruction of cortical neuronal processes (Fig. 7G and H). The observed difference between EGFP and muGFP fluorescence was reproducible over three cleared whole brains, with replicate images presented in Supplementary Figure 8. 2 mm sections were also cut from one mouse brain and subjected to Sca*l*e clearing, with EGFP and muGFP transduced neurons exhibiting similar fluorescence intensities, as expected (Supplementary Figure 9).

## Discussion

GFP is a small, folded protein and the entirety of the β-barrel structure is required for chromophore formation and fluorescence^24,25^. Structural unfolding attenuates fluorescence, and thus increased stability of the protein structure under denaturing conditions is desirable for applications such as imaging cleared tissue samples, which require harsh chemical treatment. Our previous study described the engineering of usGFP to increase thermal stability in the presence of SDS^18^. This variant contained two mutations, Q69L and N164Y, and the apparent melting temperature of the evolved mutant was increased by 20 °C in the presence of SDS compared to the parent FP, sfGFP. In this study, we solved the crystal structure of usGFP to gain insight into the structural basis of increased stability. Our biophysical characterisation showed that both sfGFP and usGFP dimerise significantly in solution, which is undesirable for some applications. Thus, we rationally engineered a variant designed to disrupt the dimeric interface and showed that this mutant, muGFP, was monomeric in solution, even at concentrations of approximately 10 mg mL^−1^, and retained structural stability similar to usGFP in the presence of detergent and heat.

Examination of the mutated residues that confer the structural stability of usGFP and muGFP showed that residue 69 lies within the core of the β-barrel, adjacent to and in contact with the chromophore of the protein. Despite its proximity to the chromophore, the mutation of this residue has little effect on the fluorescence excitation or emission properties of the protein^18^. Mutation of the polar residue, glutamine, to leucine at this position most likely improves hydrophobic packing with residues F64, V150, I152, and L201 that surround it, leading to increased rigidity in the structure^26,27^. A further interesting result of this mutation was the exclusion of a water molecule present in the interior of the protein and hydrogen bonded to Q69 in sfGFP (Fig. 1B and E). Residues V150 and I152 are located on β-strands 7 and L201 is located on β-strand 10 in the β-barrel. Previous work has suggested that water exchange between the interior of the β-barrel and the solvent occurs through a ‘hole in the barrel’ between strands 7 and 10^26^. Increased hydrophobicity in this pocket and local rigidity could reduce water exchange, further contributing to structural stability.

The stabilising effect of the N164Y mutation, on the surface of the β-barrel, was less clear. The hydrogen bonding network surrounding this residue was different in each of sfGFP, usGFP, muGFP and EGFP^28^. However, it is clear that the tyrosine side chain was able to form more extensive hydrogen bonding networks with surrounding residues, which may contribute to structural stability. It is interesting to note that position 164 is within β-strand 8 of the barrel and directly adjacent to β-strand 7, which forms part of the dynamic structure described above. Thus, it is possible that the increased propensity for inter-side chain hydrogen bonding at the surface conferred by Y164 may further stabilise this region of the structure.

The crystal structures of usGFP and sfGFP suggested that these GFP variants form dimers. Previous studies investigating the oligomerisation of FPs have employed techniques such as SDS-PAGE, and SEC to characterise the oligomeric state of fluorescent proteins *in vitro*^7,29,30^. However, these techniques can be inadequate when investigating subtle self-association. Diffusion and dilution effects in polyacrylamide gels and SEC columns can give inaccurate measurements on the oligomeric state of the FP. In this study, we have conducted a comprehensive biophysical characterisation of GFP constructs and showed that usGFP and sfGFP showed a strong tendency to dimerise. Introducing a single mutation at the dimer interface of usGFP reduced self-association to levels below detection at the concentrations we examined. These observations are in line with previous work showing that introducing positively charged residues surrounding the dimer interface significantly reduced self-association in the commonly used variants CFP, YFP or GFP^7^.

Critically, introduction of the single mutation F223D to disrupt the dimer had no effect on the fluorescence characteristics of muGFP, and only a slight effect on the stability of muGFP with respect to usGFP (Fig. 5). In combination with the observation that the affinities of the self-association of sfGFP and usGFP were very similar, this supports the hypothesis that the Q69L and N164Y mutations contributed directly to increased stability, rather than indirectly through stabilisation of quaternary structure. Use of a validated monomeric FP is particularly important for FP fusion studies, where proteins of interest are fused to FPs to enable visualisation, localisation or even functional characterisation of the fusion. The self-associating tendency of sfGFP, usGFP and EGFP could lead to undesired or artefactual effects upon fusion to particular proteins in live animals, especially membrane proteins.

Thorough characterization of the absorption and fluorescence characteristics of EGFP, sfGFP, usGFP and muGFP in the absence and presence of SDS revealed some interesting differences. usGFP and muGFP have lower quantum yields, but higher extinction coefficients than EGFP and sfGFP. This results in usGFP and muGFP displaying higher brightness, especially in the presence of SDS, which lowers the quantum yields of EGFP and sfGFP. SDS treatment also resulted in an increase in the absorption and excitation of EGFP and sfGFP at 400 nm, which was not seen for usGFP or muGFP. The 400 nm excitation peak of GFP is associated with the neutral, hydroxyl state of the Tyr66 side chain in the chromophore of GFP, whereas the ionized phenolate state of Tyr66 is responsible for the 470–490 nm excitation peak^25^. The S65T mutation found in EGFP and sfGFP promotes a hydrogen bonding network that stabilizes the anionic state of the Tyr66 side chain and thus leads to a predominant excitation peak at 490 nm^25^. The increase in the 400 nm peak upon SDS treatment of sfGFP and EGFP suggests that the hydrogen bonding network around the chromophore of these proteins is disrupted, probably leading to the reduced performance of these FPs in SDS compared to usGFP and muGFP.

The increased stability and monomeric behaviour of muGFP was predicted to be advantageous for imaging techniques that subject biological samples to potentially denaturing chemical environments, such as tissue clearing methods. A major goal of modern neuroscience is to map the networks of connections made by individual neurons onto the architecture of the entire brain. The combination of genetically encoded FPs, immunofluorescence staining and optical microscopy of thin brain sections has provided great insights into the structure and function of rodent brains. However, it remains challenging to use these approaches to probe connections that span the large three dimensional volume of the brain. Sectioning of the brain allows optical access but destroys connections; whereas, increased sample thickness has the potential to hamper microscopic imaging due to lipids in cellular membranes scattering light, thus limiting resolution and imaging depth^11^. Tissue clearing techniques alleviate this problem by removing lipids, while maintaining the structural integrity and microanatomy of the tissue, and equilibrating the refractive index throughout the sample. When expressed in cultured mammalian cells, muGFP and EGFP exhibited similar loss of fluorescence upon fixation with 4% PFA (Supplementary Figure 7), indicating that muGFP can resist the first step of tissue clearing. Modern tissue clearing techniques subsequently use either dehydrating solvents or detergents to remove lipids, both of which can denature, or quench FPs. Much effort has been invested into modifying clearing methods to retain high FP fluorescence, with methods such as Sca*l*e resulting in high retention of FP fluorescence after clearing^12^. muGFP exhibited slightly higher fluorescence to EGFP in rAAV transduced neurons in sections of fixed mouse cortex (Fig. 6), and after clearing with Sca*l*e (Supplementary Figure 9), demonstrating that muGFP is well expressed *in vivo* and is equally suitable to tissue clearing methods already optimised for existing FPs.

For detailed mapping and cellular phenotyping across networks, FP labelling must be combined with immunofluorescent labelling. While immunofluorescent labelling can be conducted on thick sections with Sca*l*eS, with similar preservation of FP fluorescence to Sca*l*e^31^, immunolabelling of Sca*l*eS-cleared whole mouse brains has not been reported. Immunofluorescence labelling in cleared tissues has been successful with techniques such as 3DISCO and CLARITY, but the agents used for clearing in these methods result in reduced FP fluorescence^13,15,17^. In 3DISCO solvents are used to dehydrate the tissue and strip away lipids, which leads to tissue shrinkage^11^. In CLARITY however, tissues are fixed and crosslinked to a hydrogel in aqueous solution to retain native tissue morphology. Lipids are then removed by electrophoresis in SDS at 37 °C–50 °C for CLARITY^14^, or incubation for weeks in SDS at 37 °C–55 °C for passive CLARITY^15^. While some GFP variants exhibit relatively high stability in SDS^32^, long incubation periods in SDS at high temperatures will quench most GFP variants, and researchers must compromise on the speed and level of clarification required when performing CLARITY on FP-expressing samples^15^. With muGFP labelled mouse brains, however, such a compromise was not required, with muGFP labelled neurons exhibiting bright fluorescence in 3 mm sections cleared for 3 weeks at 55 °C (Fig. 6), or whole brains cleared for 5 weeks at 55 °C (Fig. 7). Conversely, fluorescence of EGFP transduced neurons after the same treatments was strongly attenuated, demonstrating that the high stability of muGFP in SDS makes this FP a superior probe for CLARITY. The strong muGFP staining in CLARITY-cleared mouse brains enabled the imaging of deep confocal stacks for 3D reconstruction, which was not possible with the very low fluorescence exhibited in the EGFP transduced samples.

This work provides a novel and versatile FP, muGFP, which is highly stable and remains monomeric at high concentrations, making it an ideal candidate for use in many different biological and biophysical applications. Importantly, the monomeric nature of muGFP offers a critical advantage for applications where the localisation and proximity of tagged proteins is assessed, including in widely used FRET studies, or when protein localisation may be influenced by oligomerisation. Resistance to denaturation by heat, SDS, and common fixatives will be beneficial where strongly denaturing conditions are required, and will greatly expand the versatility and applications of the emerging whole-organ imaging technique, CLARITY.

## Methods

### Protein expression and purification

Residues 1–238 of sfGFP or usGFP with an N-terminal 6 × His tag were cloned into a custom vector based on pQE30 using BamHI and HindIII restriction sites. The F223D mutation was introduced into the pQE30-usGFP construct using the PrimeStar Mutagenesis kit (Takara, Shiga, Japan) and confirmed by sequencing. Proteins were expressed in E.coli BL21(DE3) cultured in LB medium at 20 °C for 16 h. Cells were harvested by centrifugation and resuspended in buffer (25 mM Tris-HCl, pH 7.5, 150 mM NaCl, 0.6% 3-[(3-cholamidopropyl)dimethylammonio]-1-propanesulfonate (CHAPS)) before lysis by sonication. Clarified lysate was applied to 3 mL TALON (Clontech) resin pre-equilibrated in 25 mM Tris, pH 7.5 and 150 mM NaCl and incubated at 4 °C for 1 h with gentle rocking. Resin was washed with 5 column volumes of wash buffer (25 mM Tris pH 7.5, 150 mM NaCl and 10 mM imidazole) and then eluted with elution buffer (equilibration buffer with 250 mM imidazole). GFP containing fractions were concentrated and applied to a Superdex™ 200 10/300 GL column (GE Healthcare Life Sciences) and eluted with 25 mM Tris, pH 7.5 and 150 mM NaCl. Purified proteins were concentrated for use in further analyses.

### Sedimentation velocity analytical ultracentrifugation

Sedimentation velocity experiments were carried out in a Beckman-Coulter XL-I ultracentrifuge with UV-Vis scanning optics. 380 µL sample and 400 µL reference (20 mM Tris pH 7.5 and 150 mM NaCl) solutions were loaded into 12 mm charcoal-epon double sector cells with quartz windows and mounted in an An-60Ti 4-hole rotor. All GFP variants were centrifuged at concentrations of 1.3 mg mL^−1^, 0.65 mg mL^−1^ and 0.33 mg mL^−1^ at 50,000 rpm (201,600 × *g*) and 20 °C. Radial absorbance data were collected at 280 nm in continuous mode and were fitted to a continuous sedimentation coefficient distribution [c(s)] model using SEDFIT^33^ and converted to standardised [c(s_20_,_w_)] distributions. SEDNTERP^34^ was used to calculate buffer density (1.005 g mL^−1^), buffer viscosity (1.021 cp), and the partial specific volumes of sfGFP (0.732 g mL^−1^), usGFP (0.733 mL g-1), and muGFP (0.732 mL g^−1^).

### Crystallisation of usGFP and muGFP

usGFP and muGFP were crystallised using the sitting drop vapour diffusion method. Crystals of usGFP were obtained from crystallant containing 0.2 M sodium nitrate, 20% (w/v) PEG 3350, 0.1 M bis-tris propane, pH 6.5 at 8 °C. Crystals were cryoprotected by brief soaking in crystallant supplemented with 7.5% v/v glycerol and 7.5% v/v ethylene glycol before flash cooling in liquid nitrogen. Crystals of muGFP were obtained from crystallant containing 0.2 M sodium chloride, 22% w/v PEG 8000, 4% v/v acetone, 0.1 M phosphate-citrate buffer pH 3.7 at 8 °C. Crystals were flash cooled with no additional cryoprotectant. X-ray diffraction data were collected at 100 K using the microfocus macromolecular crystallography (MX2) beam line of the Australian Synchrotron under the control of the BluIce software package^35^.

### Structure determination and refinement of usGFP and muGFP

Diffraction data were indexed and integrated using the *XDS* package^36^, followed by analysis using *POINTLESS*^37^ and merging using *AIMLESS*^38^ from the *CCP4* suite^39^. Initial phase estimates were obtained by molecular replacement using *PHASER*^40^. Molecular replacement for usGFP was performed using the sfGFP structure (PDB ID 2B3P^21^); as the search model. Molecular replacement for muGFP was performed using the refined coordinates for usGFP as the search model. Structures were submitted to three cycles of simulated annealing using *PHENIX*^41^ at an early stage of the refinement to minimise model bias. Structure refinement was carried out using *REFMAC5*^42^ with iterative model building and addition of solvent performed using *COOT*^43^. Data processing and refinement statistics are shown in Table 1.

### Small-angle X-ray scattering

SAXS data were collected at the SAXS/WAXS beam line at the Australian Synchrotron using the method described previously^44^. Briefly, 50 µL purified GFP at approximately 50 mg mL^−1^ was loaded onto an in-line Superdex 75 10/300 GL size exclusion column (GE Healthcare) pre-equilibrated with 25 mM Tris pH 7.5 and 150 mM NaCl. The column was run at a flow rate of 0.2 ml min^−1^ and eluted directly into a 1.5 mm quartz capillary for data collection. 800 images (5 s exposures) were collected during elution with a Pilatus 1 M detector at a distance of 2.6 m from the capillary, giving a *q* range of 0.005 to 0.3 Å^−1^ where *q* is the magnitude of the momentum-transfer vector and *q* = (4πsinθ)/λ where the scattering angle is 2θ and λ is the X-ray wavelength (1.0322 Å). Radial averaging, normalisation and background subtraction were conducted using *SCATTERBRAIN* (Australian Synchrotron). 6 images (30 s exposure) at the elution peak were averaged for each sample, data were analysed using *ATSAS*^45^, and Guinier plots were linear for s·Rg < 1.3 (Supplementary Figure 4). The theoretical scattering curves for the GFP variants were calculated from the refined crystal structure coordinates using *CRYSOL*^46^. Experimental data were fitted to theoretical scattering curves calculated from the refined usGFP structure coordinates, as this was the most complete structural model.

### FP characterisation

Absorption and fluorescence spectra of purified FPs were measured using a CLARIOstar plate reader (BMG Labtech, Ortenberg, Germany) with samples in clear bottom, black, non-binding 96 well plates (Greiner Bio One, Kremsmünster, Austria). Protein concentrations were determined using a Direct Detect spectrometer (Millipore) and absorbance measurements at 280 nm. Thermostability measurements were conducted as previously described^18^. Briefly, purified proteins were diluted to 10 µg mL^−1^ in 100 mM NaCl, 1% SDS, 50 mM Tris-HCl pH 7.5. Each protein was aliquoted into 96 well PCR plates (100 µL per well), and the samples were heated at specified temperatures using a gradient PCR thermocycler for 30 min and cooled to 10 °C. Plates were placed on ice, samples were transferred to black non-binding 96 well plates (Greiner one, Kremsmünster, Austria) and the residual fluorescence measured in a POLARstar OMEGA plate reader (BMG Labtech, Ortenberg, Germany) with excitation at 488/12 nm and emission at 520 nm. Fluorescence intensities were normalised to samples heated at 95 °C and unheated at 4 °C as 0% and 100% max fluorescence, respectively. Apparent melting temperatures (T_m_) were determined by fitting the data to Boltzmann sigmoidal functions with Graphpad Prism 6. T_m_ values indicated are the mean and SEM of three independent denaturation experiments.

### Fluorescence in the presence of fixatives

sfGFP, usGFP, or muGFP were subcloned into pcDNA3.1+ (Life Technologies, Mulgrave, Australia) and transfected into HEK293 cells plated onto poly-lysine coated Nunc™ 96-well black-walled plates (Thermo Fisher Scientific, Victoria, Australia) using FuGENE^®^ HD reagent (Promega, Sydney, Australia), according to the manufacturer’s instructions. GFP protein was transiently expressed for 48 h and cells were treated with PBS or fixed for 20 min at 4 °C with paraformaldehyde (PFA) freshly prepared from 16% concentrated formaldehyde ampules (Thermo Fisher Scientific, Aus.), or PFA combined with common immunohistochemistry fixatives: gluteraldehyde (GA; 5% from 25% EM grade solution, Electron Microscopy Sciences, PA, USA) or 50% methanol (Me). Cells were washed 3 times with cold PBS before imaging.

GFP expression in mammalian cells was imaged before and after fixation using the Operetta® High Content Imaging System (Perkin Elmer, USA). Images for each experiment were acquired on different days with independent transfections, from six locations across the well with a 20xNA objective in wide field mode using bright-field and standard GFP filter settings (ex. 460–490 nm and em. 500–550 nm). The mean GFP intensity was obtained for cells greater than 10 μm in size and greater than 200 fluorescence units from a 16-bit image. The mean fluorescence intensity range was between 2000–4000 units per cell, and data expressed as a mean (± sem) percentage of unfixed, PBS-treated cells. Where fixation led to the complete loss of fluorescence, a region of interest was assigned a background value of 200 mean fluorescent units.

### Whole mouse brain clearing and imaging

The muGFP encoding gene was cloned into a rAAV vector (pAM-DCA-EcoRI-EGFP^47^), by replacing the EGFP gene, making pAM-DCA-EcoRI-muGFP. The pAM-DCA-EcoRI-EGFP and pAM-DCA-EcoRI-muGFP viral vectors were packaged into rAAV mosaic serotype 1/2 capsids, and the resultant rAAV1/2 preparations were harvested, purified, and the viral titres assessed as described previously^47^. The combination of the CMV enhancer/chicken β-actin promoter with the AAV1/2 serotype has been previously shown to be highly effective for specific neuronal targeting^48^. Injection of rAAV1/2-EGFP and rAAV1/2-muGFP was performed as described previously^49^. Mice at postnatal age 4–6 weeks were anaesthetised with isoflurane (Delvet, Seven Hills, NSW, Australia). A total of 100 nL virus was injected in the primary somatosensory cortex at 0.5 mm, 1 mm and 1.5 mm from pia, and brains were harvested after two weeks.

Uncleared/Sca*l*e: mice were deeply anaesthetised with sodium pentobarbitone (100 mg/kg; Virbac, Milperra, NSW, Australia) and transcardially perfused with 30 mL 0.1 M phosphate buffer (PB) followed by 25 mL 4% PFA in PB. Brains were postfixed for two days and sectioned with a vibratome. 100 μm sections were collected for the uncleared control, and a 2 mm section was cut from the same brain for Sca*l*eA2 clearing. Uncleared control samples were mounted in Antifade Gold (Thermofisher). Sca*l*eA2 samples were placed in Sca*l*eA2 medium (Olympus) and incubated for one week at 4 °C with daily medium changes.

CLARITY: mice were deeply anaesthetised as above and placed on ice. Mice were then perfused transcardially with 30 mL ice-cold phosphate buffered saline (PBS) followed by 25 mL ice-cold CLARITY hydrogel^14^. Whole brains were rapidly extracted and immediately submerged in 25 mL ice-cold hydrogel solution and post-fixed for 1 week in the dark. To prepare 3 mm-thick coronal blocks of mouse brain for clearing of sectioned brains, brains were cut into 3 mm-thick blocks using a mouse brain matrix. Hydrogel polymerization was then initiated for both whole brains and 3 mm sections by increasing the hydrogel solution temperature to 37 °C for ∼3 h. Embedded tissue was gently extracted from the gel, followed by 2 × 24 h washes in 50 ml CLARITY clearing solution (4% SDS and 0.2 mM borate, both Sigma Aldrich, St. Louis, Missouri, USA; pH 8.5) at room temperature to wash-out excess monomers, PFA and initiator. Whole-brains and 3 mm thick sections were incubated (55 °C, shaking at 0.25 g) in 2 L or 1 L of CLARITY clearing solution, respectively, and CLARITY clearing solution was replaced weekly (for 4–5 weeks for whole brains and 3 weeks for 3 mm brain sections). Whole brains and 3 mm brain sections were rinsed in PBS with 0.1% Tween-20 (Sigma Aldrich) for several days and then immersed in 80% glycerol for 24 h prior to imaging.

Quantification of the fluorescence intensities of EGFP and muGFP in pre- and post-cleared tissue sections was achieved by importing microscopy data from two mice for each sample group into Bitplane Imaris (version 8.4.1). Somata were detected using the Imaris FilamentTracer module and underwent subsequent mean sum intensity measurement with the Imaris MeasurementPro module. The FilamentTracer module detects and quantifies background fluorescence so that it can be excluded in filament tracing. Thus, background fluorescence subtraction was not necessary. In addition, background fluorescence levels of samples were manually confirmed to be comparable prior to completing filament detection and fluorescence quantification. 1.4 mm confocal stacks were acquired with 8 µm steps, using a Zeiss LSM 780 confocal microscope (Carl Zeiss AG, Oberkochen, Germany) using a 20x/0.8 air lens, or a 20x/1.0 NA water immersion objective with Sca*l*eA2 medium (Sca*l*e) or 80% glycerol (CLARITY) as the immersion medium. Stacks (Fig. 7E and F) are coloured in an arbitrary way to differentiate between the bottom and top of the stack.

All animal studies were performed in accordance with the Prevention of Cruelty to Animals Act (2004), under the guidelines of the National Health and Medical Research Council Code of Practice for the Care and Use of Animals for Experimental Purposes in Australia (2013) and approved by The Florey Animal Ethics Committee. All efforts were made to minimize animal suffering and reduce the number of animals used. Animal studies are reported in compliance with the ARRIVE guidelines.

### Data availability

Coordinates and structure factors for usGFP and muGFP have been deposited in the PDB with accession codes 5JZK and 5JZL, respectively.

## Electronic supplementary material

Supplementary Information 
