# Supplementary material for: A Novel Ultra-Stable, Monomeric Green Fluorescent Protein For Direct Volumetric Imaging of Whole Organs Using CLARITY
Source: Sci Rep. 2018 Jan 12;8:667. doi: 10.1038/s41598-017-18045-y (PMC5766548; doi:10.1038/s41598-017-18045-y)
Supplement: Supplementary file 1 — Supplementary Information [file 41598_2017_18045_MOESM1_ESM.doc]

**Supplementary information for:**

**A novel ultra-stable, monomeric green fluorescent protein for direct volumetric imaging of whole organs using CLARITY**

Daniel J. Scott^1,2^, Natalie J. Gunn^1,3^, Kelvin J. Yong^1,2^, Verena C. Wimmer^1^, Nicholas A. Veldhuis^4,5^, Leesa M. Challis^1^, Mouna Haidar^1^, Steven Petrou^1^, Ross A.D. Bathgate^1,2^ & Michael D.W. Griffin^2,6^

**Affiliations**

1. The Florey Institute of Neuroscience and Mental Health, Parkville, Victoria, 3052, Australia

2.Department of Biochemistry and Molecular Biology, Bio21 Molecular Science and Biotechnology Institute, University of Melbourne, Parkville, Victoria 3010, Australia

3. IBM Research Australia, Carlton, Victoria 3053, Australia

4. Monash Institute of Pharmaceutical Sciences, Monash University, Parkville, Victoria 3052, Australia

5. ARC Centre of Excellence in Convergent Bio-Nano Science and Technology, Monash University, Parkville, Victoria, 3052, Australia

6. Bio21 Molecular Science and Biotechnology Institute, University of Melbourne, Parkville, Victoria 3010, Australia

This supplementary material comprises:

**Supplementary Figures 1 – 9**


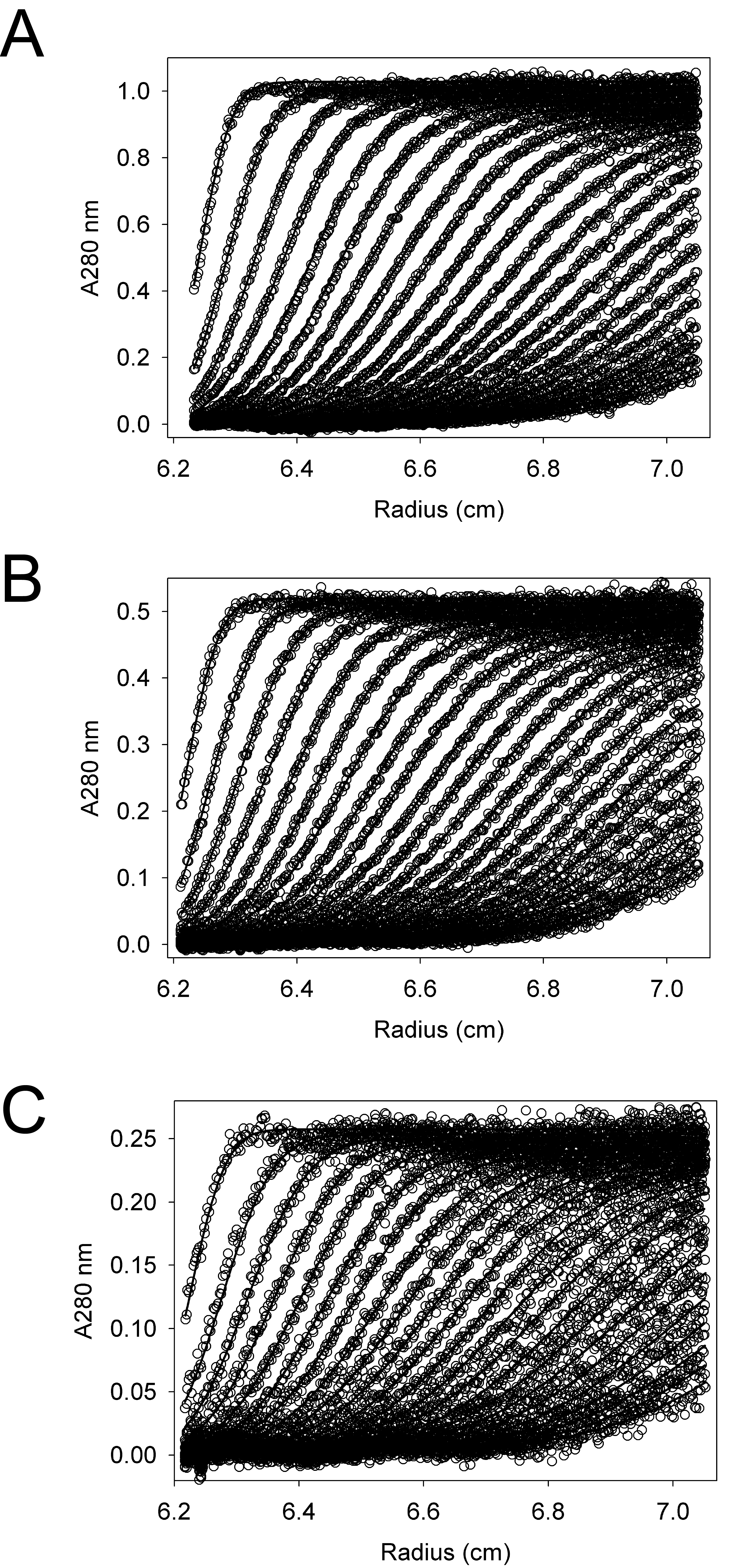


**Supplementary Figure 1**. **Sedimentation velocity analysis of usGFP at three concentrations.** Absorbance was measured at 280 nm as a function of radial position (cm). Raw data (open circles) were fitted to a c(s) distribution (solid lines) and experiments were conducted at concentrations of 1.3 mg mL^-1^ (A), 0.65 mg mL^-1^ (B) and 0.33 mg mL^-1^ (C). The c(s) distribution of usGFP is shown in Figure 2A.


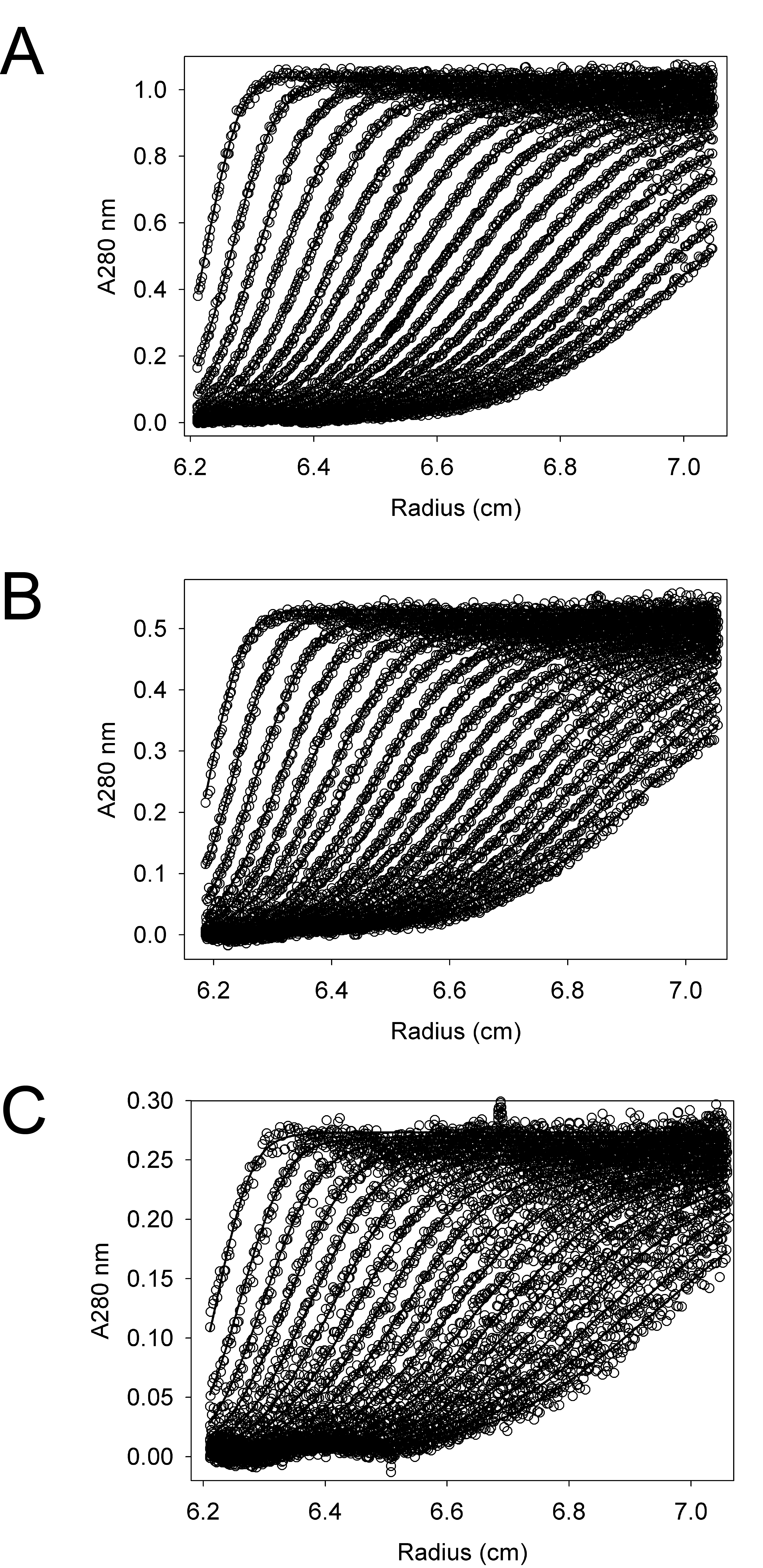


**Supplementary Figure 2**. **Sedimentation velocity analysis of sfGFP at three concentrations.** Absorbance was measured at 280 nm as a function of radial position (cm). Raw data (open circles) were fitted to a c(s) distribution (solid lines) and experiments were conducted at concentrations of 1.3 mg mL^-1^ (A), 0.65 mg mL^-1^ (B) and 0.33 mg mL^-1^ (C). The c(s) distribution of sfGFP is shown in Figure 2B.

**
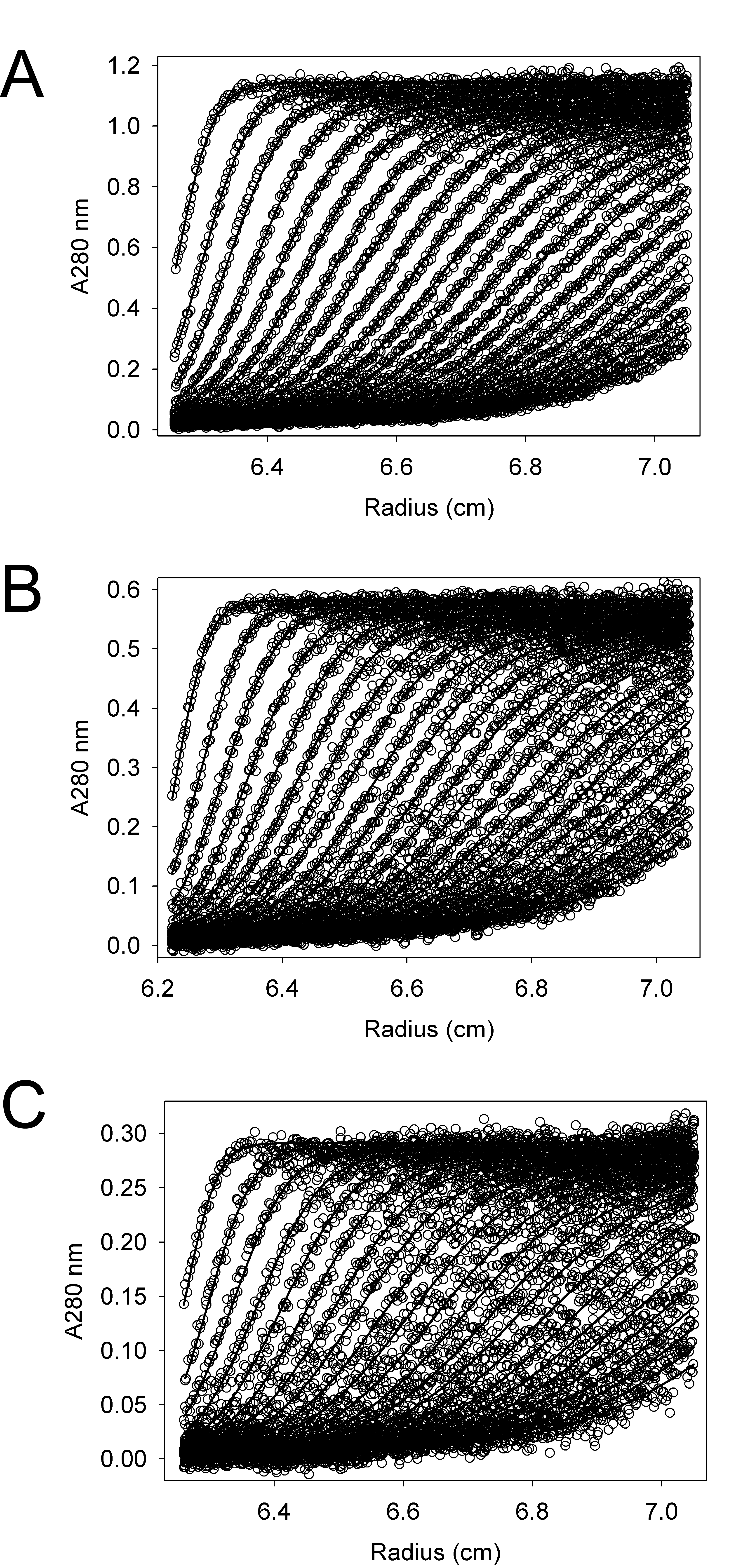
**

**Supplementary Figure 3**. **Sedimentation velocity analysis of EGFP at three concentrations.** Absorbance was measured at 280 nm as a function of radial position (cm). Raw data (open circles) were fitted to a c(s) distribution (solid lines) and experiments were conducted at concentrations of 1.3 mg mL^-1^ (A), 0.65 mg mL^-1^ (B) and 0.33 mg mL^-1^ (C). The c(s) distribution of EGFP is shown in Figure 2C.


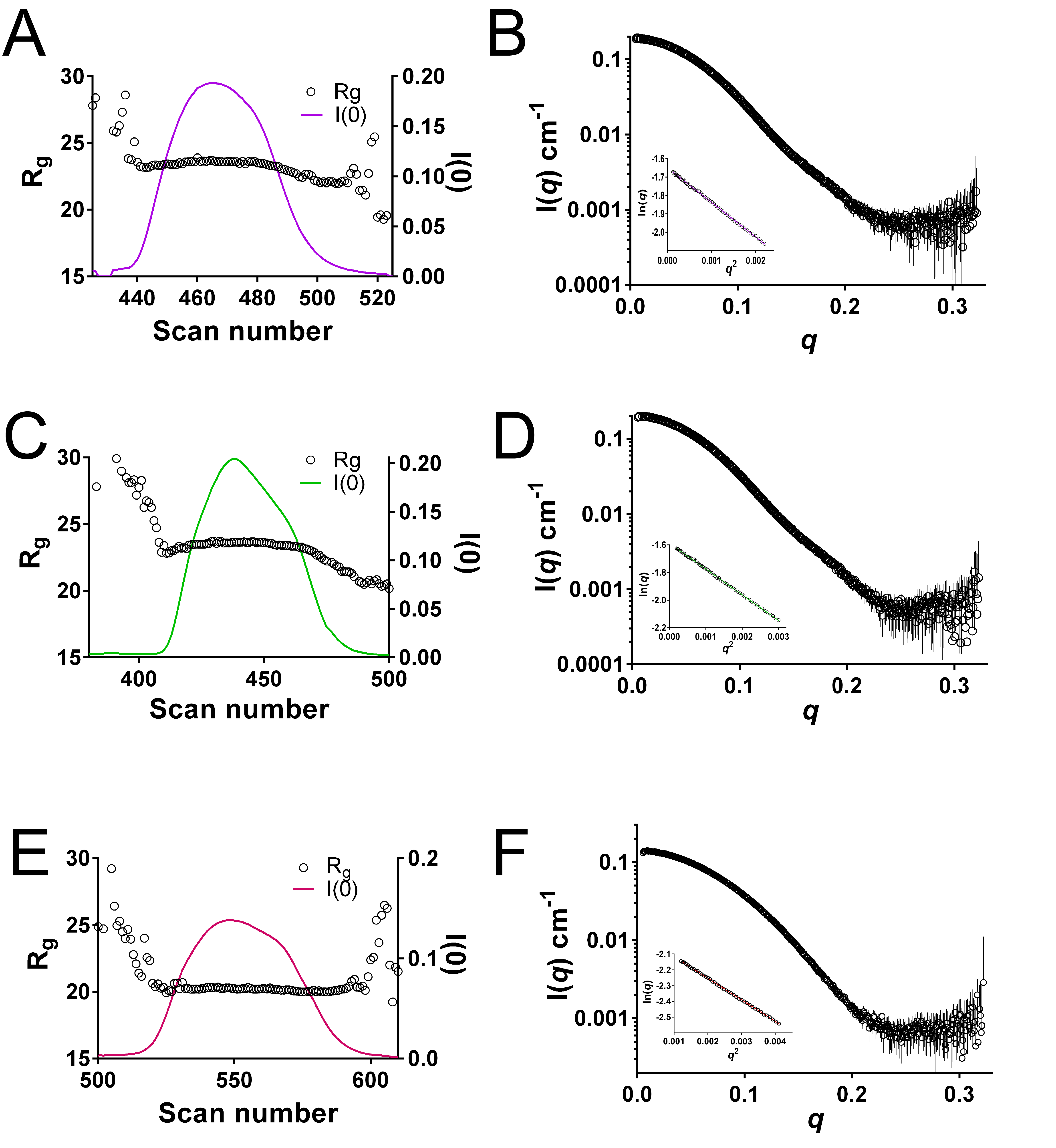


**Supplementary Figure 4**. **SAXS analysis of usGFP (A-B), sfGFP (C-D) and muGFP (E-F).** A, C and E. I(0) values (solid line) for individual images across the elution peak with R_g_ values (open circles) plotted for each image. B, D and F. Averaged scattering profile across the elution peak. *Inset* Guinier profiles for each plot.


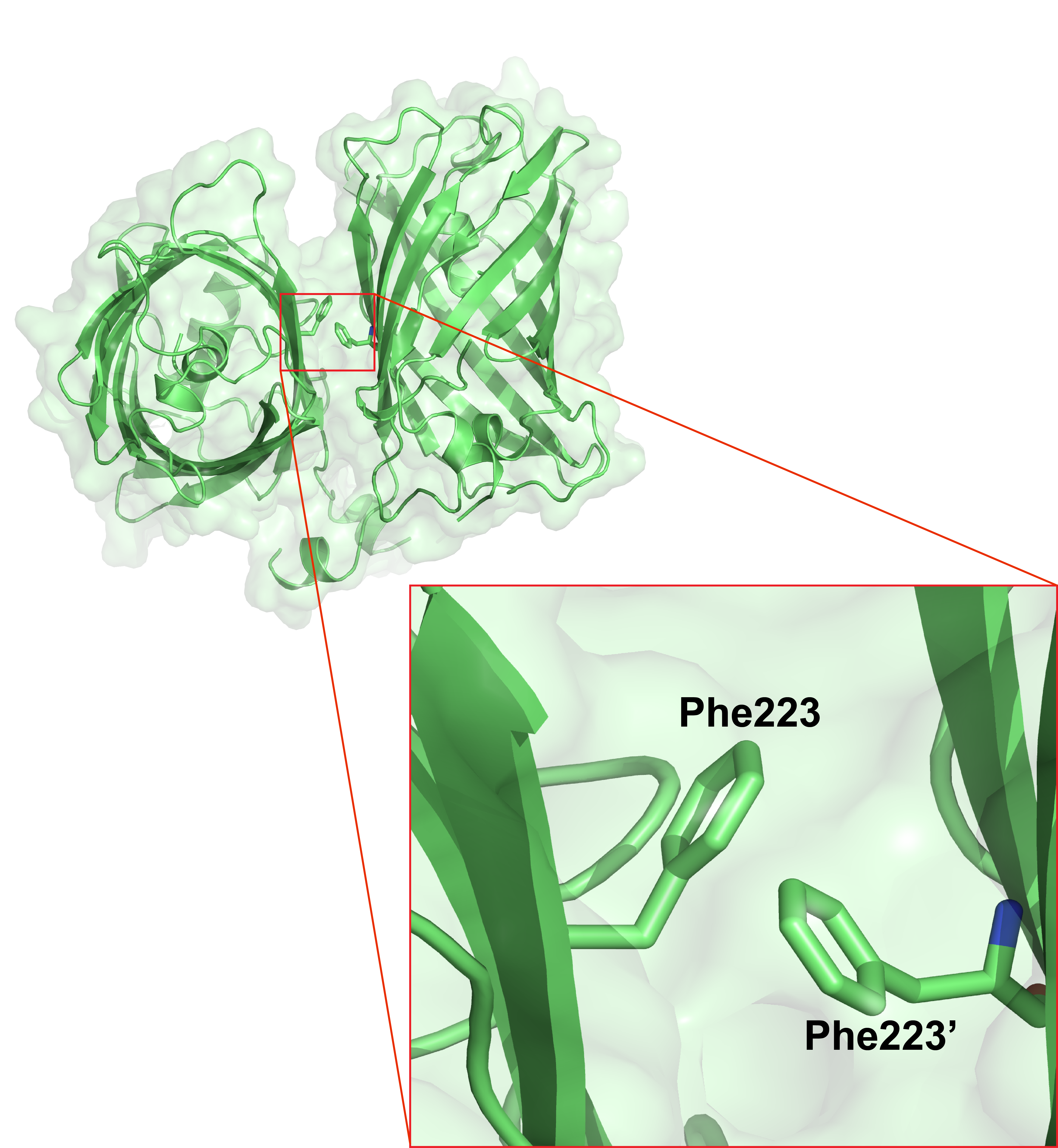


**Supplementary Figure 5.** **Crystal structure of usGFP showing phenylalanine 223 at the interface of between the two molecules in the asymmetric unit.** This residue was mutated to aspartate in muGFP.


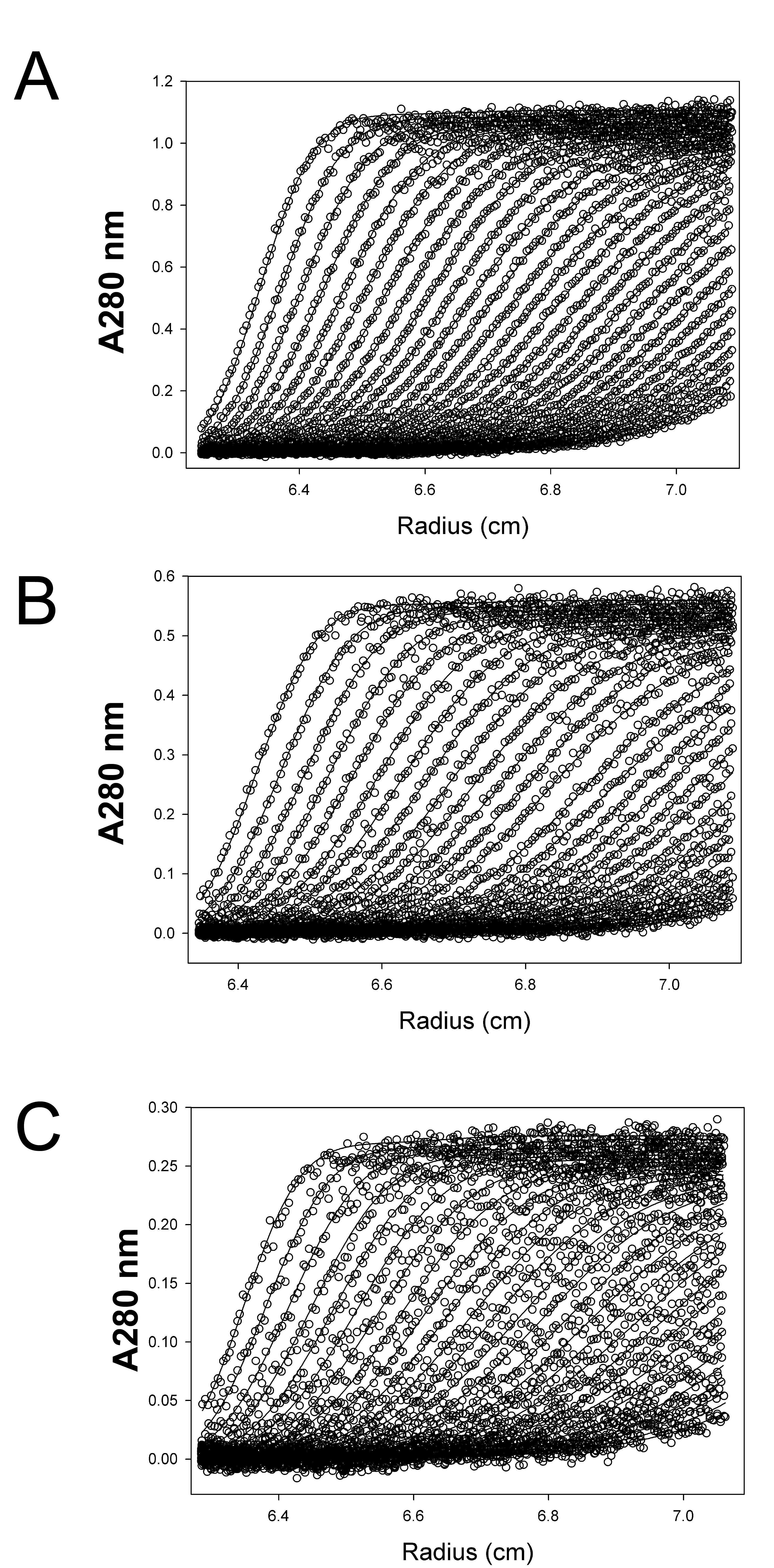


**Supplementary Figure 6**. **Sedimentation velocity analysis of muGFP at three concentrations.** Absorbance was measured at 280 nm as a function of radial position (cm). Raw data (open circles) were fitted to a c(s) distribution (solid lines) and experiments were conducted at concentrations of 1.3 mg mL^-1^ (A), 0.65 mg mL^-1^ (B) and 0.33 mg mL^-1^ (C). The c(s) distribution of muGFP is shown in Figure 5A.


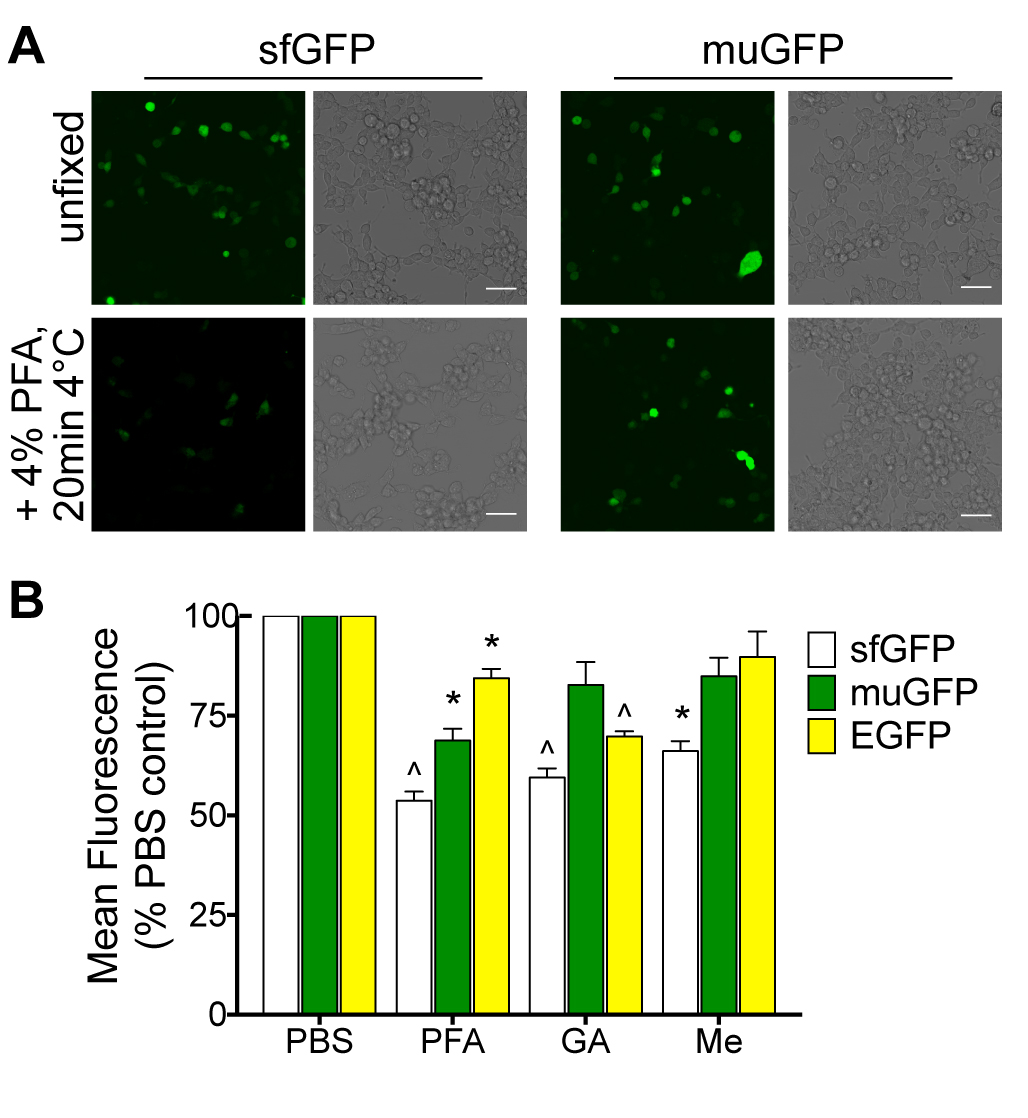


**Supplementary Figure 7.** **Expression of stabilised GFP variants in mammalian cells.** **A**. GFP Fluorescence (green channel) and bright-field images of HEK293 expressing sfGFP or muGFP before or after fixation with 4% PFA at 4°C for 20min. **B**. Mean fluorescence intensity of HEK expressing GFP variants was compared following cell treatment with freshly prepared 4% PFA, 4% PFA with 5% glutaraldehyde (GA) or 50% methanol in PBS (Me). Values are expressed in relative fluorescence units as a percentage of mean fluorescence intensity for unfixed control cells (PBS). N = 3 experiments, >1000 cells per experiment; *p<0.05; ^p<0.01; # p<0.001 by one-way ANOVA with Dunnett’s post-hoc test relative to PBS control. Scale bar = 50 μm.


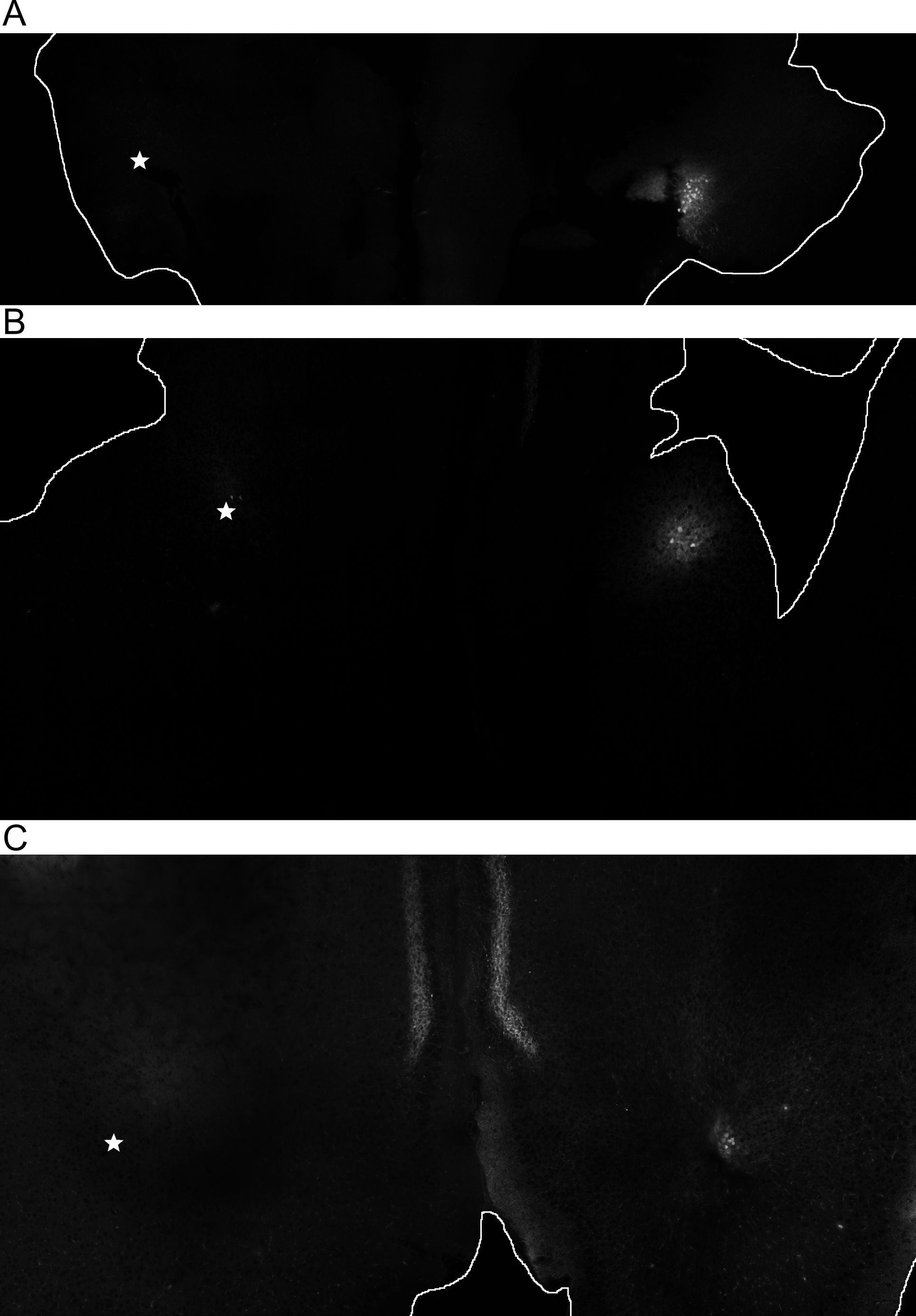


**Supplementary Figure 8**. **Confocal imaging of replicate whole mouse brains expressing EGFP and muGFP.** Low magnification confocal images across both injection sites in three whole CLARITY-cleared mouse brains (A-B). The EGFP virus injection sites are indicated with the star, on the left hemispheres, whereas muGFP virus injection sites are clearly visible based on cell fluorescence within the right hemispheres. Tissue boundaries are indicated with the white lines. The maximum projection confocal stacks presented in Figure 7 were generated from the brain in panel B. Some thalamic staining was observed in the third brain (C), which probably resulted in the muGFP virus backpropagating from the injection site.


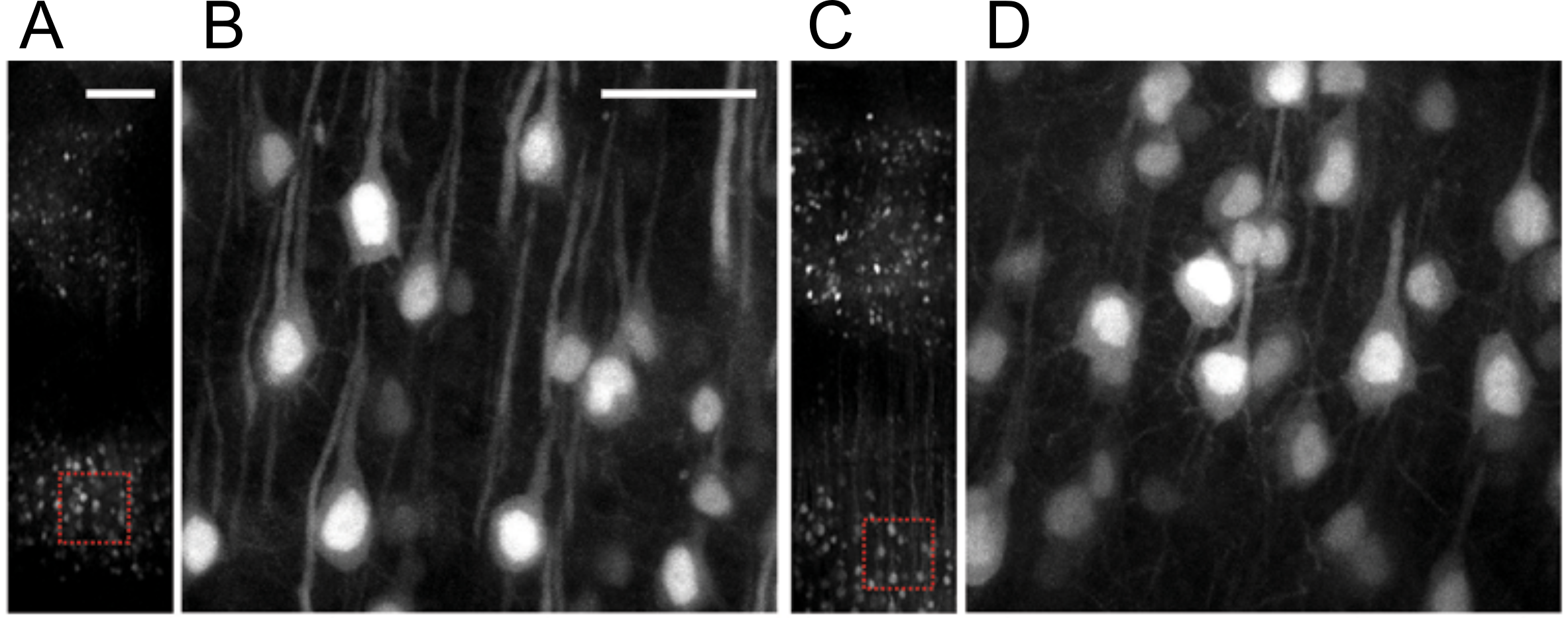


**Supplementary Figure 9**. **Confocal imaging of 2 mm sections of a mouse brain expressing EGFP and muGFP cleared with Scale.** Neurons of the primary somatosensory cortex were virally transduced with EGFP in the left hemisphere (A-B) and muGFP in the right hemisphere (C-D). Low magnification maximum projections of confocal image stacks are presented in A and C, with the red dashed squares indicating where higher magnification images were taken (B and D). Scalebars indicate 200 µm in A and C, 50 µm in B and D. Scale clearing was performed on sections from one brain.
